# TANDEM ZINC‐FINGER/PLUS3 integrates light signaling and flowering regulatory pathways at the chromatin level

**DOI:** 10.1111/nph.70213

**Published:** 2025-05-12

**Authors:** Giorgio Perrella, Elisa Vellutini, Allan Beveridge, Graham Hamilton, Pawel Herzyk, Eirini Kaiserli

**Affiliations:** ^1^ School of Molecular Biosciences, College of Medical, Veterinary and Life Sciences University of Glasgow Bower Building Glasgow G12 8QQ UK; ^2^ Department of Biosciences University of Milan Via Giovanni Celoria 26 20133 Milan Italy

**Keywords:** Arabidopsis, chromatin, flowering, histone modifications, Light, transcription

## Abstract

- Environmental and endogenous stimuli determine plant developmental transitions including flowering through multiple signaling cascades. Although the key activators and repressors of flowering initiation are defined, the components and mechanisms integrating light signaling and flowering pathways are not fully established. This study investigates the role of TANDEM ZINC‐FINGER/PLUS3 (TZP), a light‐integrating transcriptional regulator, to elucidate how light cues influence the epigenetic regulation of flowering in *Arabidopsis thaliana*.
- To dissect the molecular function of TZP, this study employed a combination of genetics, RNA sequencing, chromatin immunoprecipitation sequencing and phenotypic assays. These approaches were used to determine TZP's genomic binding sites, its downstream gene targets and its influence on flowering time and chromatin modifications. TANDEM ZINC‐FINGER/PLUS3 was found to directly associate with the promoter regions of chromatin‐modifying genes, including *FLOWERING LOCUS D* (a histone H3K4 demethylase) and histone deacetylase 6 (a histone deacetylase). This regulation promotes a chromatin environment that represses FLOWERING LOCUS C (*FLC*) transcription, thereby accelerating flowering. TANDEM ZINC‐FINGER/PLUS3 thus functions upstream of multiple pathways integrating photoperiodic and autonomous floral cues.
- TANDEM ZINC‐FINGER/PLUS3 mediates crosstalk between light signaling and flowering pathways by modulating chromatin structure at the *FLC* locus. This provides a mechanistic framework for understanding how environmental signals dynamically influence epigenetic regulation of developmental transitions.

## Introduction

Light is a major environmental factor that fine‐tunes different stages of plant development, starting from seed germination and de‐etiolation to the transition from vegetative to reproductive development during flowering initiation (Franklin, 2009; Perrella *et al*., 2020; Patitaki *et al*., 2022). Environmental cues, including day length, circadian rhythms and temperature, regulate flowering through different pathways such as vernalization (prolonged cold exposure), photoperiodic and hormone signaling (Reeves & Coupland, 2000; Fernandez *et al*., 2016). In Arabidopsis, the signaling outputs from distinct flowering cascades are integrated by two major components: FLOWERING LOCUS T (FT) and CONSTANS (CO). FLOWERING LOCUS T is the main florigen whose expression is directly induced by the transcription factor CO (Nogueira *et al*., 2024; Romero *et al*., 2024). CONSTANS is a transcription factor that contains a CCT motif and two B‐box type zinc‐finger domains (Nogueira *et al*., 2024; Romero *et al*., 2024). SUPPRESSOR OF OVEREXPRESSION OF CONSTANS 1 (SOC1) is a downstream‐flowering integrator mainly expressed in developing leaves and shoot apical meristems (Lee & Lee, 2010). SUPPRESSOR OF OVEREXPRESSION OF CONSTANS 1 expression is also upregulated by CO through FT, while it is repressed by a protein complex consisting of FLOWERING LOCUS C (FLC) and SHORT VEGETATIVE PHASE (Mateos *et al*., 2015). In addition to the photoperiodic and vernalization control, Arabidopsis flowering initiation is regulated by the autonomous pathway, which comprises a combination of RNA processing and epigenetic events controlling the floral repressor FLC (Wu *et al*., 2020). FLOWERING LOCUS C encodes a MADS box transcription factor that binds to the *FT* promoter and represses its expression (Michaels & Amasino, 1999; Sheldon *et al*., 2000; Whittaker & Dean, 2017; Madrid *et al*., 2021). This effect of FLC is dose‐dependent, and therefore, FLC works as a rheostat that determines flowering initiation in Arabidopsis in response to external cues (Simpson *et al*., 1999; Simpson, 2004). FLOWERING LOCUS C itself is regulated by a suite of sophisticated epigenetic, transcriptional and posttranscriptional mechanisms that are highly sensitive to temperature (Whittaker & Dean, 2017). Thus, the autonomous and vernalization pathways promote flowering by facilitating plant responsiveness to the environment. However, there is very limited information on whether environmental stimuli other than temperature, in particular light, influence *FLC* expression and ultimately its action.

TANDEM ZINC‐FINGER/PLUS3 (TZP) was originally identified as a growth‐promoting factor by quantitative trait locus mapping using Arabidopsis ecotypes (Loudet *et al*., 2008). Subsequent studies demonstrated that TZP functions as a transcriptional regulator of hypocotyl elongation and flowering initiation in response to specific light regimes (Kaiserli *et al*., 2015; Huang *et al*., 2016; Perrella *et al*., 2018; Zhang *et al*., 2018; Fang *et al*., 2022; Li *et al*., 2022; Feng *et al*., 2024). Independent studies have revealed that TZP associates with multiprotein nuclear complexes containing light, warm temperature and clock components (such as the evening complex) and directly interacts with the red/far‐red light receptors phytochrome (phy) phyA and phyB in nuclear bodies formed through liquid–liquid phase separation (Kaiserli *et al*., 2015; Huang *et al*., 2016; Perrella *et al*., 2018; Zhang *et al*., 2018; Fang *et al*., 2022; Li *et al*., 2022; Feng *et al*., 2024). Chromatin immunoprecipitation (ChIP) assays and gene expression analyses have shown that TZP can associate with growth‐ and flowering‐promoting loci, including the *FT* and *CO* promoter regions, and thereby inducing their expression in response to a long‐day (LD) photoperiod (Kaiserli *et al*., 2015).

Here, we uncover a role for TZP in controlling flowering initiation in Arabidopsis at the chromatin level by modulating *FLC* expression. By combining transcriptomics (RNA‐seq) and whole‐genome ChIP assays (ChIP‐seq) and genetic analysis, we show that TZP controls the expression of several photoperiod – independent flowering genes, including *FLC*. In addition, we reveal that TZP associates with promoter regions and induces the expression of flowering integrators including *SOC1*, as well as the chromatin remodelers histone deacetylase (*HDA*) (Yu *et al*., 2011) and histone demethylase *FLOWERING LOCUS D* (*FLD*) (Chou & Yang, 1998; Yu *et al*., 2011). Through *HDA6* and *FLD* induction, TZP controls the histone acetylation and methylation status of the *FLC* locus, which in turn is required for fine‐tuning *FLC* expression. Altogether, our study positions TZP at the crossroads integrating distinct flowering pathways and provides a new role for light signaling components in modulating the epigenetic status of key regulatory genes in plant development.

## Materials and Methods

### Plant material and growth conditions

*Arabidopsis thaliana* wild‐type (WT) and all mutants (*tzp‐1*, *co‐10*, *axe1‐5*, *ft‐10* and *flc‐3*) and transgenic lines are in Col‐0 and have been previously described (Michaels & Amasino, 2001; Probst *et al*., 2004; Kaiserli *et al*., 2015; Riboni *et al*., 2016). Overexpressing (OX) TZP/*flc*‐3 and OX TZP/*ft‐10* were generated by genetic crossing. For all the flowering experiments, Arabidopsis seeds were stratified on soil and kept in the dark at 4°C for 3 d before growing in the conditions described in the figure legends. For RNA and chromatin isolation, seeds were surface‐sterilized and sown on 0.8% agar plates containing ½ Murashige and Skoog salts in the absence of sucrose. Plates were stratified in the dark for 3 d, and plants were grown as described in the figure legends. For ChIP‐seq and RNA‐seq, tissue was collected on Day 12 under LD conditions at the time points indicated in the figure legends.

### Flowering measurements

Flowering time experiments were performed in Fitotron® growth rooms under the indicated photoperiod (LDs, 16‐h light /8‐h darkness) at 50–70 μmol m^−2^ s^−1^ white light. Flowering time was monitored by counting the total number of rosette leaves on the day of bolting (appearance of the first flower bud with a stem of 1–2 cm) and by calculating the number of days after germination at the time of bolting. One‐way analysis of variance (ANOVA) with Tukey's multiple comparison *post hoc* test was performed.

### RNA extraction and quantitative RT‐PCR

Total RNA was extracted using RNAeasy plant mini kit (Qiagen). cDNA was synthesized with the QuantiTect reverse transcription kit (Qiagen) following the manufacturer's instructions. Quantitative reverse transcription polymerase chain reaction (RT‐PCR) was performed using a StepOnePlus real‐time machine (Thermofisher) with Brilliant III UltraFast SYBR QPCR Master Mix (Agilent). *C*_t_ values and standards were analyzed as previously described (Kaiserli *et al*., 2015). Reactions were performed on four technical replicates and three independent biological replicates. The following cycling conditions were used for quantitative PCR: 2 min at 95°C, 40 cycles of 3 s at 95°C and 30 s at 59.5°C. Melting curve analysis from 60 to 90°C was performed to monitor the specificity of the amplification. Normalization of the quantitative real‐time PCR data was calculated by geometric averaging of the internal reference gene IRON–SULFUR CLUSTER ASSEMBLY PROTEIN 1 (*ISU1*) (Kaiserli *et al*., 2015; Petersen *et al*., 2017). One‐way ANOVA with Tukey's multiple comparison *post hoc* test was performed.

### Chromatin immunoprecipitation

Chromatin immunoprecipitation assays were performed as described previously with minor modifications (Perrella *et al*., 2018, 2024). A bioruptor sonicator (B01020001; Diagenode, Morris Avenue, NJ, USA) was used to shear the chromatin using the following settings: 40 cycles, 30‐s ON and 30‐s OFF at high power. For immunoprecipitation, the following antibodies were used: anti‐GFP (AbCam, Ab290), anti‐H3K9K14Ac and anti‐H3K4me3 (pAb‐005‐50 and pAb‐003‐50; Diagenode). Chromatin immunoprecipitation–quantitative polymerase chain reaction (ChIP‐qPCR) was performed at the following cycles: 95°C × 3 min, 95°C × 3 s, 59.5°C × 30 s (45 cycles), 95°C × 1 min and 60°C × 30 s (melting curve). Relative enrichment was calculated as described previously (Kaiserli *et al*., 2015; Perrella *et al*., 2018). Reactions were performed on four technical replicates and three independent biological replicates. Chromatin immunoprecipitation samples from the three experimental replicates were pooled and subjected to Illumina sequencing (ChIP‐seq). A fourth additional replicate was performed to verify TZP association on selected loci by ChIP‐qPCR. A complete list of primers used for genotyping, quantitative reverse transcription polymerase chain reaction (qRT‐PCR), cloning and ChIP‐qPCR is presented in Supplementary Information Table S1.

### Next‐generation sequencing analysis

Next‐generation sequencing analysis of the RNA and ChIP samples was carried out in the Glasgow Polyomics Facility (University of Glasgow). For RNA sequencing, libraries were prepared from total RNA using the Illumina TruSeq Stranded mRNA Sample Preparation Kit. Libraries were sequenced in 75‐base paired‐end mode on the Illumina NextSeq 500 platform. Raw sequence reads were trimmed for contaminating sequence adapters and poor‐quality bases using the program Cutadapt (https://doi.org/10.14806/ej.17.1.200). Bases with an average Phred score lower than 15 were trimmed. Reads that were trimmed to < 54 bases were discarded. The quality of the reads was checked using the Fastqc program (http://www.bioinformatics.babraham.ac.uk/projects/fastqc/) before and after trimming. The reads were ‘pseudo‐aligned’ to the transcriptome using the program Kallisto (Bray *et al*., 2016). The differential expression for the analysis groups was assessed using the Bioconductor package DESeq2 (Love *et al*., 2014). Chromatin immunoprecipitation sequencing libraries were sequenced in 75‐base paired‐end mode on the Illumina NextSeq 500 platform. Raw sequence reads were trimmed for contaminating sequence adapters and poor‐quality bases using the program Cutadapt. Bases with an average Phred score lower than 15 were trimmed. Reads that were trimmed to less than 54 bases were discarded. The quality of the reads was checked using the Fastqc program (http://www.bioinformatics.babraham.ac.uk/projects/fastqc/) before and after trimming. The reads were aligned to the reference genome using Bowtie2 (Langmead & Salzberg, 2012). Peaks were called using the Sicer program (Zang *et al*., 2009) and the peaks annotated using the Bioconductor R package ChIPpeakAnno (Zhu *et al*., 2010). Interpolation and Venn diagram were created using the online tool Venny 2.1 (bioinfogp.cnb.csic.es/tools/venny/). All RNA‐seq, ChIP‐seq, Gene Ontology (GO) and comparison datasets are listed in Tables [Link], [Link], [Link], [Link], [Link], [Link], [Link], [Link], [Link], [Link], [Link], [Link], [Link], [Link], [Link], [Link], [Link].

## Results

### TZP promotes flowering through multiple pathways

**Fig. 1:**
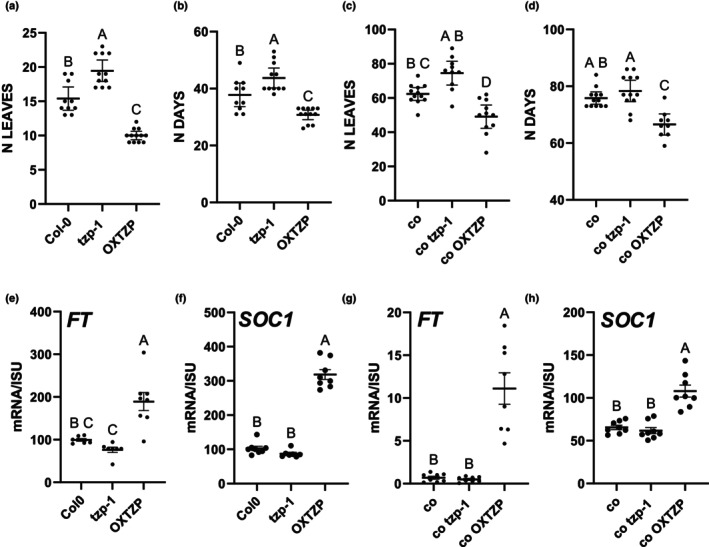
TANDEM ZINC‐FINGER/PLUS3 (TZP) promotes flowering initiation partially independent of CONSTANS in Arabidopsis. (a, b) Phenotypic characterization of the number of leaves (a) and the number of days (b) at bolting in Col‐0, *tzp‐1* and OXTZP. (c, d) Phenotypic characterization of the number of leaves (c) and the number of days (d) at bolting in genetic crosses between CONSTANS (*co*) and *tzp‐1* or OXTZP. Data are representative of three independent biological replicates. Plants were grown under long‐day (LD = 16 h : 8 h, light : dark) photoperiodic conditions. Data are represented as mean ± 95% confidence interval (*n* = 10–12 plants). One‐way analysis of variance (ANOVA) with Tukey's multiple comparison *post hoc* test was performed. (e–h) Reverse transcription quantitative polymerase chain reaction analysis of *FT* (e–g) and *SOC1* (f–h), transcript levels normalized to the housekeeping gene *ISU1*. Tissue was isolated at ZT 15 on Day 12 under LD white light (50 μmol m^−2^ s^−1^). Wild‐type was used as a reference. Data shown are represented as mean ± SE and are representative of four technical replicates out of two biological replicates. One‐way ANOVA with Tukey's multiple comparison *post hoc* test was performed. Upper case letters indicate statistically significant differences among the groups. Groups that share the same letter are not significantly different from each other, while groups with different letters. FT, FLOWERING LOCUS T; ISU1, IRON–SULFUR CLUSTER ASSEMBLY PROTEIN 1; N Leaves, no. of leaves; OXTZP, overexpression of TANDEM ZINC‐FINGER/PLUS3; SOC1, SUPPRESSOR OF OVEREXPRESSION OF CONSTANS 1; ZT, Zeitgeber Time.

Previous reports showed that TZP promotes flowering initiation under LD photoperiodic conditions (16 h : 8 h, 22°C, light :dark) (Kaiserli *et al*., 2015). This was attributed to an increase in *FT* expression in seedlings OX TZP in Col‐0 and Bay‐0 (Kaiserli *et al*., 2015). To further investigate the role of TZP in controlling flowering initiation, we monitored flowering time in the *tzp* mutant (*tzp‐1*) (Fig. 1a,b) that was characterized previously (Perrella *et al*., 2018). As anticipated, *tzp‐1* exhibited a mildly delayed but statistically significant flowering phenotype compared with WT (Col‐0) as measured by the number of leaves and days at bolting (Figs 1a,b, S1A). Molecular analysis also showed a modest but not statistically significant reduction in the transcript levels of the florigen *FT* as well as the flowering integrator *SOC1* in *tzp‐1* compared with WT. Conversely, the expression of both *FT* and *SOC1* was upregulated in OXTZP compared with that in WT, directly correlating with the flowering phenotype (Fig. 1e–h).

CONSTANS is a major transcriptional regulator of *FT*; therefore, to assess the genetic relationship between TZP and CO, we introgressed *tzp‐1* and OXTZP in the *co* mutant background. Flowering experiments revealed that, as previously reported, *co* exhibits delayed flowering compared with WT (Putterill *et al*., 1995). In addition, *co tzp‐1* produced a higher number of leaves at the time of flowering initiation than *co* (Fig. 1c,d), but the number of days at bolting was not significantly different between *co* and *co tzp‐1*. On the contrary, overexpression of *TZP* in *co* (*co* OXTZP) resulted in a significantly earlier flowering initiation phenotype than in *co* (Fig. 1c,d). Gene expression analysis of *FT* and *SOC1* revealed an upregulation of both genes in *co OXTZP* compared with that in *co*, whereas no difference was observed in *co tzp‐1* (Fig. 1e–h). These data suggest that TZP controls flowering by modulating *FT* and *SOC1* expression through multiple flowering pathways or partially independent of *CO*.

### TZP controls the expression of flowering components of the photoperiodic and autonomous pathways

**Fig. 2:**
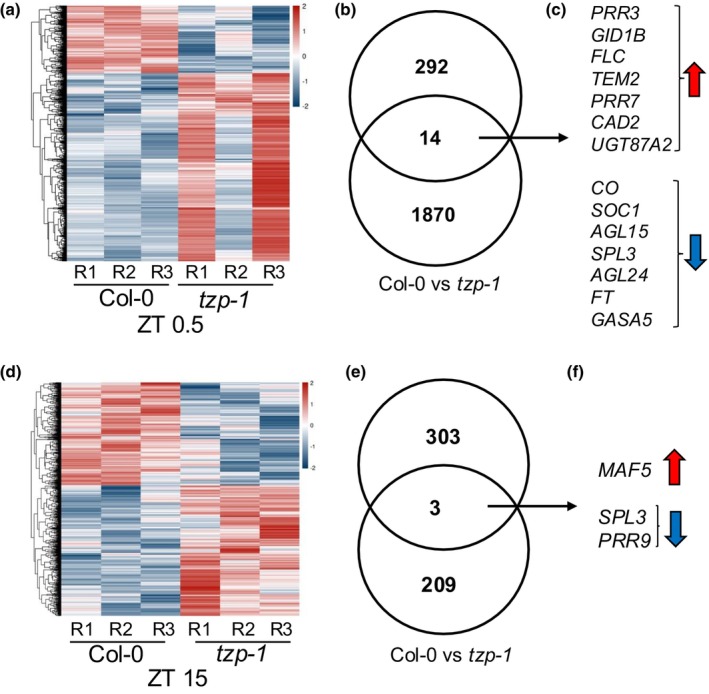
RNA‐seq identifies novel TANDEM ZINC‐FINGER/PLUS3 (TZP)‐regulated targets involved in flowering regulation in Arabidopsis. (a, d) Hierarchical clustering of differentially expressed genes (DEGs) between the indicated genotypes. Heatmaps depict the expression profile of differentially regulated genes between Col‐0 and *tzp‐1* at ZT 0.5 (a) and ZT 15 (d) (*P*‐value < 0.05; the color shades depict the *Z*‐score expression values, with blue as lower expression and red as higher; R1,2,3 depict the results from three biological replicates). Differentially expressed genes identified when comparing Col‐0 and *tzp‐1* at ZT 0.5 (b, c) and ZT 15 (e, f) were intersected with the list of flowering genes from the FLOweRing Interacting database (FLOR‐ID; Bouche *et al*., 2016). (b, e) Venn diagrams showing the overlap between DEGs between Col‐0 and *tzp‐1* (lower circle) with FLOR‐ID (upper circle) at ZT 0.5 (b) and at ZT 15 (e). (c, f) List of differentially regulated flowering genes from the FLOR‐ID database between Col‐0 and *tzp‐1* at ZT 0.5 (c) and at ZT 15 (f). The red up arrow indicates upregulated genes, and the blue down arrow indicates downregulated genes. Interpolation and Venn diagram were created using the online tool Venny 2.1 (bioinfogp.cnb.csic.es/tools/venny/). R, replicate; vs, versus; ZT, Zeitgeber Time.

To evaluate the genome‐wide role of TZP in regulating gene expression during flowering initiation, we performed transcriptome analysis by RNA sequencing on tissue collected from the 12‐d‐old WT, *tzp‐1* (Figs 2, S2A,B) and OXTZP (Figs S2C,D, S3) plants grown under a LD photoperiod at dawn Zeitgeber Time (ZT) 0.5 (dawn) and ZT 15 (dusk). A pair‐wise comparison between Col‐0 and *tzp‐1* revealed that the majority of differentially expressed genes (DEGs) with a *P*‐value ≤ 0.05 were observed at ZT 0.5 (1884 transcripts) (Figs 2a–c, S2A; Table S2). On the contrary, only 212 genes showed a significant difference in expression pattern at ZT 15 (Fig. 2d–f; Table S3). Gene ontology analyses using the software DAVID (Huang *et al*., 2007; da Huang *et al*., 2009a,b; Sherman *et al*., 2022) on transcripts that showed at least 1.5‐fold change difference in expression identified an overrepresentation of targets involved in transcription, response to stress and metabolic processes (Fig. S4D–I; Tables S4– S7). To further investigate the role of TZP in regulating flowering initiation at the transcriptional level, DEGs identified when comparing Col‐0 and *tzp‐1* (1884 at ZT 0.5 and 212 at ZT 15) were intersected with the FLOweRing Interacting database (FLOR‐ID) (Bouche *et al*., 2016). The Venn diagrams shown in Fig. 2b,e depict the overlap between DEGs between Col‐0 and *tzp‐1* (lower circle) with FLOR‐ID (upper circle) at ZT 0.5 (Fig. 2b) and ZT 15 (Fig. 2e). The intersection of the Venn diagram (Fig. 2b–c,e–f) provided a list of genes controlled by TZP that are involved in flowering time (14 genes at ZT 0.5 and three genes at ZT 15). A red upward‐facing arrow indicates the upregulated genes in *tzp‐1*, and a blue downward‐facing arrow for the downregulated genes at ZT 0.5 (Fig. 2c) and ZT 15 (Fig. 2f). The list of TZP‐regulated flowering components contained three key positive regulators *FT*, *CO* and *SOC1* and the negative regulator of flowering *FLC* (Fig. 2c). More specifically, *FT*, *CO* and *SOC1* were downregulated whereas *FLC* upregulated in *tzp‐1* compared with that in WT at dawn (Fig. 2c). Additional members of the MADS‐domain transcription factor family, including *AGAMOUS‐LIKE 15* (*AGL15*) and *AGL24*, known to act as floral integrators upstream of meristem identity regulators (Lee *et al*., 2008; Smaczniak *et al*., 2012) were also shown to be downregulated in the absence of functional TZP. On the contrary, the floral repressor TEMPRANILLO 2 and the circadian clock components *PSEUDO RESPONSE REGULATOR* (*PRR*) *3* and *PRR7* (Ratcliffe *et al*., 2003) were upregulated in *tzp‐1* compared with WT at dawn. The role of TZP at dusk (ZT 15) was less apparent since only three flowering components were differentially regulated in *tzp‐1* compared with WT (Fig. 2d–f). More specifically, the *FLC* paralog *MADS AFFECTING FLOWERING 2* (*MAF2*) (Ratcliffe *et al*., 2003) was upregulated, whereas *PRR9* and the transcriptional activators of flowering *SQUAMOSA PROMOTER‐BINDING PROTEIN‐LIKE 3* (*SPL3*) (Jung *et al*., 2016) were downregulated in the absence of functional *TZP* at ZT 15 (Fig. 2d–f).

**Fig. 3:**
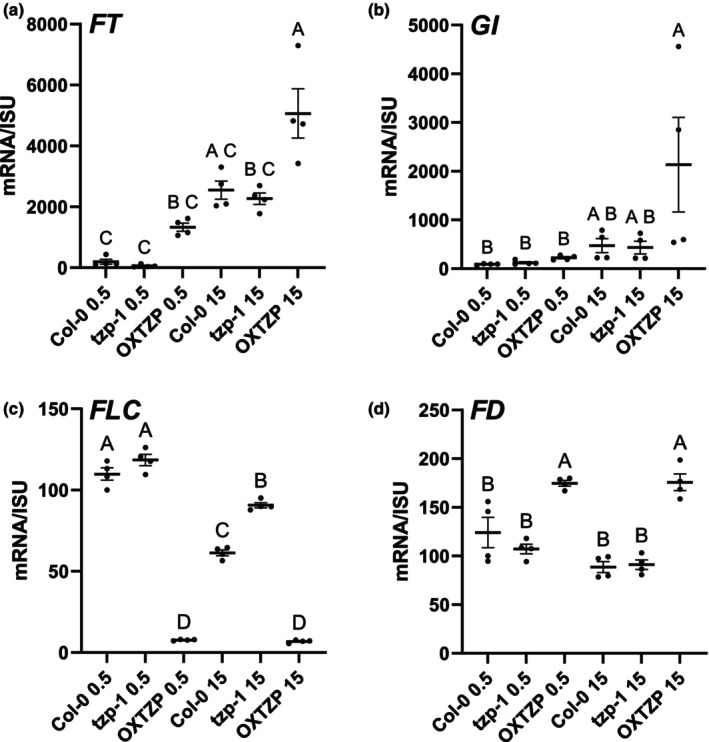
TANDEM ZINC‐FINGER/PLUS3 (TZP) regulates the expression of flowering genes operating in the photoperiodic and autonomous pathways in Arabidopsis. (a–d) Validation of selected genes identified by RNA‐seq using quantitative reverse transcription polymerase chain reaction. Plants were grown under a long‐day photoperiod (white light 50 μmol m^−2^ s^−1^). Plant tissue was collected after 12 d at ZT 0.5 and ZT 15. The expression of genes involved in flowering regulation including *FT* (a), *GI* (b), *FLC* (c) and *FD* (d) was monitored in Col‐0, *tzp‐1* and OXTZP at ZT 0.5 and ZT 15. Transcript levels were normalized with housekeeping gene *ISU1*. Col‐0 (WT) was used as a reference. Data are presented as mean ± SE. One‐way analysis of variance with Tukey's multiple comparison *post hoc* test was performed. Upper case letters indicate statistically significant differences among the groups. Groups that share the same letter are not significantly different from each other, while groups with different letters. FLC, FLOWERING LOCUS C; FT, FLOWERING LOCUS T; ISU1, IRON–SULFUR CLUSTER ASSEMBLY PROTEIN 1; OXTZP, Overexpression of TANDEM ZINC‐FINGER/PLUS3; ZT, Zeitgeber Time.

Transcriptome analysis between Col‐0 and OXTZP showed a much greater number of genes differentially regulated between the two genotypes than in *tzp‐1*. At ZT 0.5, 5210 transcripts exhibited a significant expression between Col‐0 and OXTZP, while at ZT 15, the number of DEGs decreased to 3948 (Fig. S3B,C, E,F; Tables S8– S12). Consistent with *tzp‐1*, OXTZP also showed more apparent differences at dawn rather than at dusk. Gene ontology analysis conducted by DAVID indicated that TZP‐regulated genes were classified in gene clusters including response to hormones, transcription and metabolic processes (Fig. S4J–O; Tables S13– S16). Intersecting the differentially regulated genes between OXTZP and WT with the FLOR‐ID database showed an increase in the transcript levels of positively regulating flowering components including *FT* and *SOC1* at dawn (Fig. S3B,C) oppositely to the pattern observed for *tzp‐1*, showing a decrease in *FT* and *SOC1* mRNA (Fig. 2b,c). In addition, RNA sequencing analysis revealed that the flowering‐promoting bZIP transcription factor *FD* (Jung *et al*., 2016; Gorham *et al*., 2018; Zhu *et al*., 2020) was upregulated in OXTZP (Fig. S3F). The circadian clock components *PRR3* and *PRR5* (Farre & Liu, 2013) were upregulated in OXTZP at ZT 0.5 and in *PRR7* at ZT 15, respectively (Fig. S3C,F) whereas *GIGANTEA* (*GI*) (Liu *et al*., 2024) was upregulated specifically at ZT 15 (Fig. S3F). Contrary to our findings for *tzp‐1*, *SPL3* and *SPL9* were upregulated in OXTZP, whereas *SPL3* was downregulated in *tzp‐1* (Figs S3F, 2c,f). The flowering repressor *FLC* was also found to be differentially expressed in OXTZP showing a decrease in mRNA levels compared with that in WT at both ZT 0.5 and ZT 15 (Fig. S3C,F) following an opposite trend to the upregulation that was observed in *tzp‐1* (Fig. 2c). To verify the findings from the RNA‐seq experiments on WT, *tzp‐1* and OXTZP, RT‐qPCR assays were conducted and verified the effect of TZP on the *FLC* expression pattern at both dawn and dusk (Fig. 3c), with a more pronounced regulation at ZT 15 as previously reported for *FT* (Fig. 3a; Kaiserli *et al*., 2015). On the contrary, the expression of *FD* and the evening complex components *GI* were only upregulated in OXTZP either at ZT 15 (*GI*) or at both time points (*FD*) (Fig. 3b,d). Thus, our data show that TZP acts as a positive regulator of flowering by controlling the expression levels of a diverse array of flowering integrators operating photoperiod‐dependent and photoperiod‐independent pathways.

### TZP associates with promoter regions during flowering induction

**Fig. 4:**
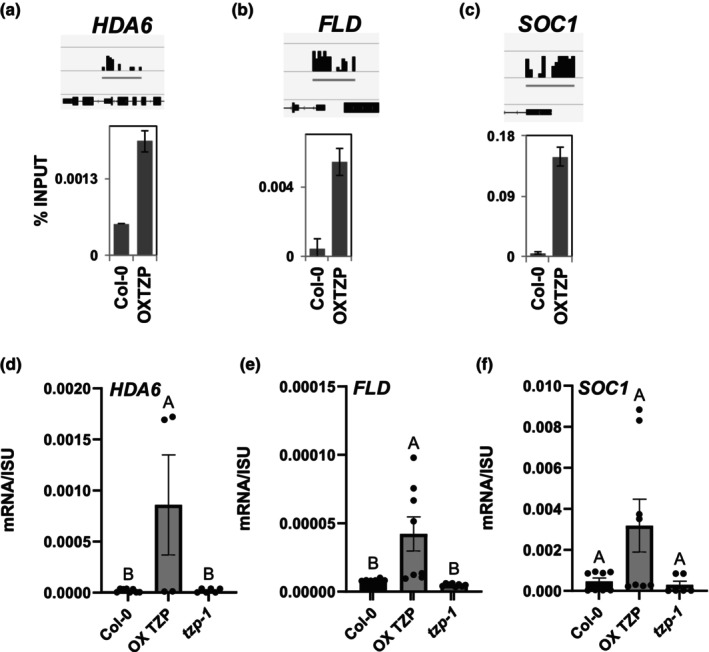
TANDEM ZINC‐FINGER/PLUS3 directly regulates the expression of chromatin regulators operating in the autonomous flowering pathway in Arabidopsis. (a–f) Validation of selected ChIP‐seq and RNA‐seq targets including *HDA6*, *FLD* and *SOC1* by chromatin immunoprecipitation–quantitative polymerase chain reaction (a–c) and reverse transcription quantitative polymerase chain reaction (d–f). Plants were grown in long‐day photoperiodic conditions (white light 50 μmol m^−2^ s^−1^) for 12 d. (d–f) Data shown are shown as mean ± SE. (*n* = 8 replicates). One‐way analysis of variance with Tukey's multiple comparison *post hoc* test was performed. Upper case letters indicate statistically significant differences among the groups. Groups that share the same letter are not significantly different from each other, while groups with different letters. FLD, FLOWERING LOCUS D; FT, FLOWERING LOCUS T; HDA6, HISTONE DEACETYLASE 6; ISU1, IRON–SULFUR CLUSTER ASSEMBLY PROTEIN 1; OXTZP, overexpression of TANDEM ZINC‐FINGER/PLUS3; SOC1, SUPPRESSOR OF OVEREXPRESSION OF CONSTANS 1.

TANDEM ZINC‐FINGER/PLUS3 has been reported to bind promoter elements of light and photoperiod‐regulated genes, including *FT* (Kaiserli *et al*., 2015; Perrella *et al*., 2018). Therefore, to identify genome‐wide TZP‐associated loci potentially involved in TZP‐controlled flowering initiation, we performed ChIP coupled with next‐generation sequencing on 12‐d‐old GFP‐tagged TZP (OXTZP) and control WT plants grown under a LD photoperiod at two different time points: ZT 0.5 and ZT 15. Relative binding‐peak distribution of ChIP‐seq data revealed that TZP associates primarily with promoters and the first exon regions at both time points, with 67% and 80% of the genomic binding sites spanning within these regions, respectively (Fig. S5; Table S17). Data analysis revealed that 92 loci were bound by TZP at both ZT 0.5 and ZT 15, while 418 and 610 loci were bound by TZP at ZT 0.5 or ZT 15, respectively (Fig. S5B). *De novo* analysis using Homer (http://homer.ucsd.edu/homer/motif/) for identifying consensus motifs enriched within the promoter‐binding peaks of TZP at ZT 0.5 and ZT 15 identified G‐box‐like elements (CACGTG) and the TATA motif required for transcriptional initiation (Fig. S5C) (Yamamoto *et al*., 2009; Liu *et al*., 2013; Ezer *et al*., 2017). Filtering of TZP‐associated target loci through FLOR‐ID revealed 16 flowering‐related genes, including *SOC1*, the autonomous pathway components *HDA6* and the histone demethylase *FLD* (Fig. S5D,E) (Chou & Yang, 1998; Yu *et al*., 2011). Moreover, TZP was found to associate with the promoters of the clock components *CIRCADIAN CLOCK‐ASSOCIATED 1* (Green & Tobin, 1999) and *LATE ELONGATED HYPOCOTYL* (Schaffer *et al*., 1998) (Fig. S5D,E). To investigate whether the association of TZP with promoter regions leads to changes in gene expression, we verified selected ChIP‐seq targets (*HDA6*, *FLD* and *SOC1*) by ChIP‐qPCR (Fig. 4a–c; Table S17). In addition, we investigated the effect of TZP binding on the transcript levels of *HDA6*, *FLD* and *SOC1* by RT‐qPCR and showed their upregulation in OXTZP and downregulation in *tzp‐1* compared with that in WT (Fig. 4d–f). Furthermore, a comparison of the differentially regulated genes between *tzp‐1* and Col‐0 as well as OXTZP and Col‐0 with the list of TZP‐associated loci identified from ChIP‐seq experiments was conducted (Fig. S5H,I; Table S18) and showed that, in addition to components operating in hormone and clock pathways, *SOC1*, a key floral integrator of photoperiod, temperature, hormones and age‐related pathways (Lee & Lee, 2010), is directly regulated by TZP and differentially expressed in *tzp‐1* and OXTZP compared with that in WT, which highlights the role of TZP in modulating flowering in response to diverse endogenous and environmental stimuli. In summary, our experiments reveal that TZP associates with promoter regions and regulates the expression of flowering genes operating at the chromatin level in response to environmentally controlled and endogenous pathways (Figs 4, S5). Notably, we revealed a direct link between TZP and the flowering integrator SOC1, proving its importance in the regulation of plant development.

### TZP negatively regulates the expression of *FLC* at the chromatin level

Data obtained from the ChIP‐seq and ChIP‐qPCR analyses showed that TZP binds to the promoter of the flowering regulator *SOC1* and the autonomous pathway chromatin‐modifying enzymes *HDA6* and *FLD* to induce their expression (Figs 4, S5E). Histone deacetylase 6 is a HDA with an established function in repressing gene expression to control leaf development and flowering (Yu *et al*., 2011; Luo *et al*., 2012; Tan *et al*., 2018). With respect to flowering time, the *hda6* mutant allele *axe1‐5* exhibits delayed flowering in response to both LD and short day (SD) photoperiodic conditions, with a more pronounced phenotype in the latter than in the former (Yu *et al*., 2011) *FLOWERING LOCUS D* is a histone demethylase that contains a SWIRM domain, known for mediating protein–protein interactions within chromatin modification complexes (Chou & Yang, 1998; He *et al*., 2003; Yu *et al*., 2011). Similar to *axe1‐5*, *fld* lines exhibit a late‐flowering phenotype in LD and SD conditions. Double‐mutant *axe1‐5 fld* lines show an even more pronounced delay in flowering than each single mutant and WT, suggesting an additive or synergistic role for the two genes (Yu *et al*., 2011). Gene expression analyses in the single and double mutants revealed an upregulation in *FLC* transcript abundance as well as in the MADS box genes *MAF4* and *MAF5* belonging to the *FLC* phylogenetic clade (Yu *et al*., 2011; Gu *et al*., 2013). Furthermore, *axe1‐5* and *fld* display global as well as locus‐dependent H3 hyperacetylation and H3 hypermethylation on *FLC*, *MAF4* and *MAF5* loci (Yu *et al*., 2011).

**Fig. 5:**
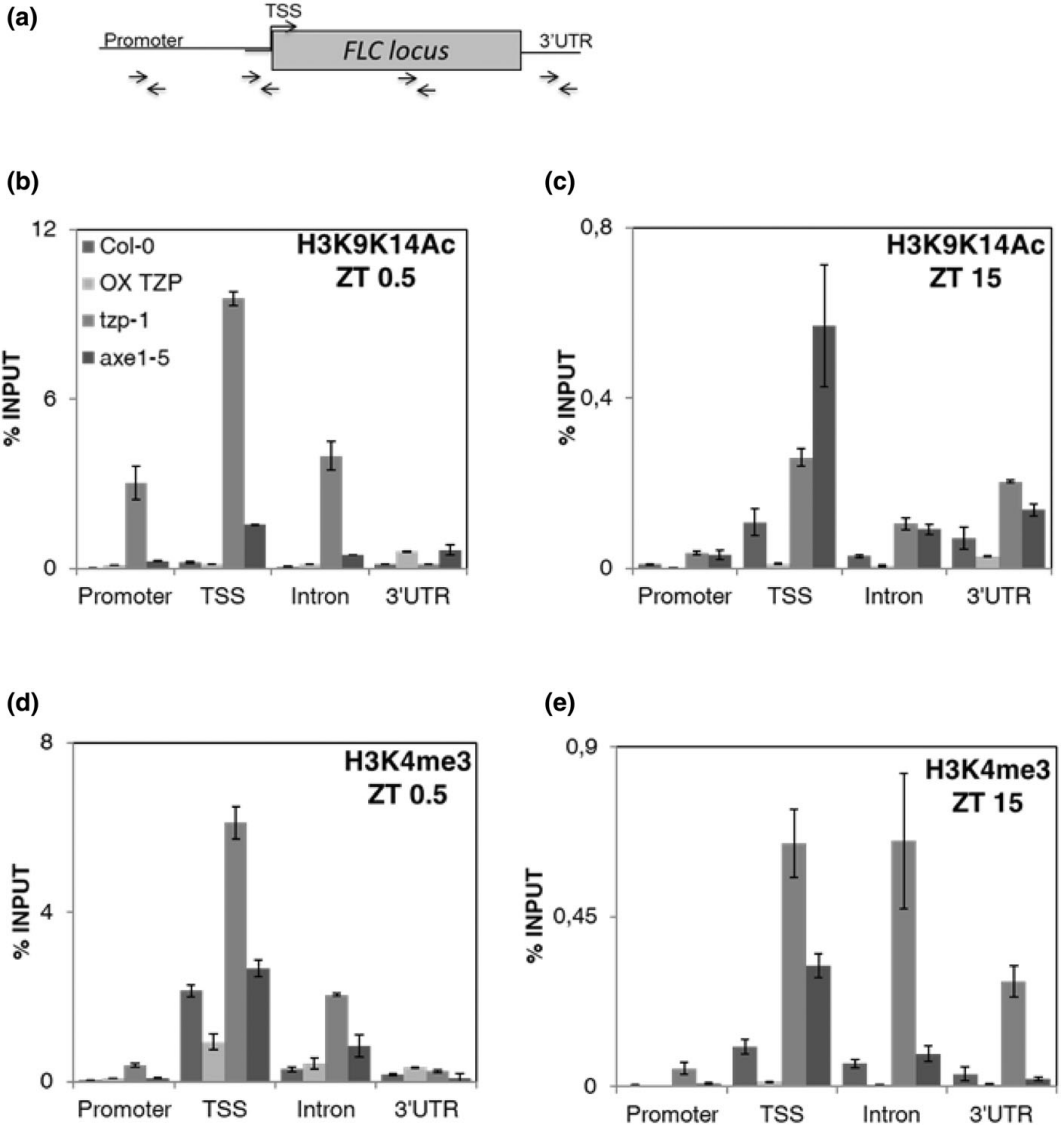
TANDEM ZINC‐FINGER/PLUS3 (TZP) regulates the acetylation and methylation status of histone tails on the Arabidopsis FLOWERING LOCUS C (*FLC*) locus. (a) Schematic representation of the *FLC* locus depicting the regions under investigation by chromatin immunoprecipitation–quantitative polymerase chain reaction. (b–d) Relative H3K9K14Ac (b–c) and H3K4me3 (d–e) enrichment in Col‐0, OX TZP, *tzp‐1* and *axe1‐5*, at ZT 0.5 (b–d) and ZT 15 (c–e), respectively. The 3′ untranslated region was used as a negative control. Seedlings were grown in white light (75 mmol m^−2^ s^−1^), and samples were harvested at ZT 0.5 and ZT 15 of Day 12. Error bars are mean ± SE (*n* = 4 technical replicates). Graphs shown are representative of two independent biological repeats. FLC, FLOWERING LOCUS C; H3K4me3, trimethylation of lysine 4 on histone H3; H3K9K14Ac, acetylation of lysines 9 and 14 on histone H3; OXTZP, overexpression of TANDEM ZINC‐FINGER/PLUS3; TSS, transcriptional start site; ZT, Zeitgeber Time.

Since TZP is a positive regulator of *HDA6* and *FLD* expression, we hypothesized that TZP is controlling *FLC* transcript abundance by influencing the acetylation and methylation status of the *FLC* promoter. To test this, we performed ChIP‐qPCR using anti‐H3K9K14Ac and H3K4me3 antibodies in WT (Col‐0), OXTZP, *tzp‐1* and *axe1‐5* lines. Our ChIP‐qPCR data revealed an increase in H3K9K14Ac acetylation on the first exon and intron of *FLC* in *axe1‐5*, agreeing with previous reports (Yu *et al*., 2011). Chromatin immunoprecipitation–quantitative polymerase chain reaction spanning different regions of the *FLC* locus (Fig. 5a) determined that primarily the promoter and TSS regions of *FLC* showed reduced H3K9K14Ac and H3K4me3 levels in OXTZP, while an increase was observed in *tzp‐1* at ZT 0.5 or ZT 15 compared with that in WT (Fig. 5b,c). As previously reported for *axe1‐5*, we observed hyperacetylation at dawn and dusk, while an increase in histone methylation was not evident compared with that in WT (Fig. 5d,e).

Thus, we provide evidence that TZP represses *FLC* transcript levels by promoting the expression of *HDA6* and *FLD*, which subsequently regulate *FLC* chromatin compaction by removing histone acetylation and methylation marks on the TSS of *FLC*. Contrary to *FLC*, no TZP‐mediated effect was observed in the H3K9K14Ac and H3K4me3 status of the TZP‐regulated flowering repressor *TEMPRANILLO 1* (*TEM1*) (Castillejo & Pelaz, 2008; Hu *et al*., 2021) (Fig. S6), suggesting that the role of TZP in modulating H3 acetylation and methylation is specific to *FLC*.

**Fig. 6:**
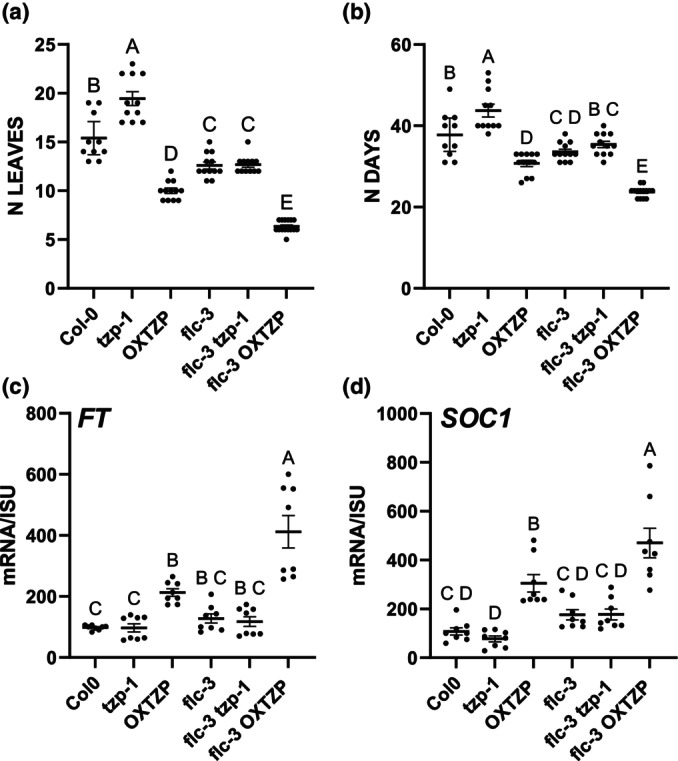
TANDEM ZINC‐FINGER/PLUS3 (TZP) overexpression enhances the early flowering phenotype of *flc* mutant in Arabidopsis. (a, b) Phenotypic characterization of flowering time (number of leaves at the time of bolting) in genetic crosses between *flc‐3* and *tzp‐1* or OXTZP. Plants were grown under long‐day (LD) 16 h : 8 h, light : dark photoperiodic conditions. Data are represented as mean ± 95% confidence interval (*n* = 10 plants). Data are representative of two biological replicates. One‐way analysis of variance (ANOVA) with Tukey's multiple comparison *post hoc* test was performed. (c, d) Reverse transcription quantitative polymerase chain reaction analysis of *FT* (c) and *SOC1* (d) transcript levels normalized to the housekeeping gene *ISU1*. Tissue was isolated at ZT 15 on Day 12 under LD white light (50 μmol m^−2^ s^−1^). Wild‐type was used as a reference. Data shown are represented as mean ± SE and are representative of technical replicates out of two biological replicates. One‐way ANOVA with Tukey's multiple comparison *post hoc* test was performed. Upper case letters indicate statistically significant differences among the groups. Groups that share the same letter are not significantly different from each other, while groups with different letters. FT, FLOWERING LOCUS T; ISU1, IRON–SULFUR CLUSTER ASSEMBLY PROTEIN 1; N Leaves, no. of leaves; OXTZP, overexpression of TANDEM ZINC‐FINGER/PLUS3; SOC1, SUPPRESSOR OF OVEREXPRESSION OF CONSTANS 1; ZT, Zeitgeber Time.

To understand the physiological relevance of TZP in controlling *FLC* expression, we performed a genetic cross between the *flc‐3* mutant in Col‐0 and *tzp‐1* or *OXTZP* and monitored flowering initiation in LD at ambient temperature. *Flc‐3* and *flc‐3 tzp‐1* exhibited earlier flowering phenotypes than WT (Fig. 6a,b). No significant difference was observed in *flc‐3 tzp‐1* compared with that in *flc‐3* (Fig. 6a,b). Interestingly, overexpression of *TZP* in *flc‐3* (OXTZP *flc‐3*) resulted in a statistically significant earlier flowering phenotype than in *flc‐3* (Fig. 6a,b). Furthermore, qRT‐PCR showed an increase in the induction of *FT* and *SOC1* transcript levels in OXTZP *flc‐3* compared with that in *flc‐3* and *WT* (Fig. 6c,d), thereby providing a molecular basis for the phenotypic data (Fig. 6a,b). Conversely, introgression of OXTZP in the *ft‐10* mutant background showed a statistically significant reduction in flowering time compared with *ft‐10* (Fig. S7A,B). *SOC1* induction was reduced in *ft‐10* and *ft‐10 tzp‐1* while we observed a small increase in *SOC1* levels in *ft‐10* OXTZP compared with that in *ft‐10* (Fig. S7C). Thus, our data indicate that the mutation in *FLC* allows a greater induction of flowering genes, suggesting that TZP action is not solely based on the *FLC* expression status, but it largely depends on the abundance and presence of *FT*.

Altogether, our findings uncover a new mechanism of TZP action, providing a link between light signaling and the autonomous pathway at the chromatin level to control flowering initiation in Arabidopsis.

## Discussion

We previously reported that TZP promotes flowering in response to LDs by binding to the promoter of *FT* and upregulating its expression in a phyB‐dependent manner (Kaiserli *et al*., 2015). To further explore the function of TZP in the transcriptional regulation of flowering, we employed a genome‐wide chromatin association study and transcriptome analysis approach. RNA sequencing and ChIP sequencing revealed that *FT* is upregulated in OXTZP, as previously reported (Kaiserli *et al*., 2015), and downregulated in *tzp‐1* (Figs 2C, S3C). Furthermore, flowering‐regulating genes including *SOC1*, *FD* and *GI* were shown to be controlled by TZP (Figs 2C, S3C,F, 3a–d). Unexpectedly, the flowering repressor FLC, which is controlled by the vernalization and autonomous pathways, was shown to be negatively regulated by TZP (Figs 2c, S3C,F). This discovery prompted us to explore a new role for TZP in integrating light signaling with flowering regulation pathways. ChIP‐seq analysis revealed that TZP associates with promoter regions and controls the expression of positive (*GI*, *SOC1*, *HDA6* and *FLD*) as well as negative (*TEM1*) regulators of flowering operating through the photoperiodic, aging, hormone, vernalization and autonomous pathways (Fig. S5) (Castillejo & Pelaz, 2008; Lee & Lee, 2010; Yu *et al*., 2011; Osnato *et al*., 2012). There are limited reports indicating a crosstalk between light signaling and autonomous flowering pathways through the interaction between phyB and PHY‐DEPENDENT LATE‐FLOWERING (PHL) or VASCULAR PLANT ONE‐ZINC‐FINGER 2, respectively (Endo *et al*., 2013; Qu *et al*., 2024). However, both mechanisms rely on the action of CO. Here, we provide evidence for a TZP‐mediated integration of photoperiodic and autonomous flowering pathways through a direct association with flowering‐regulating and chromatin‐remodeling loci.

**Fig. 7:**
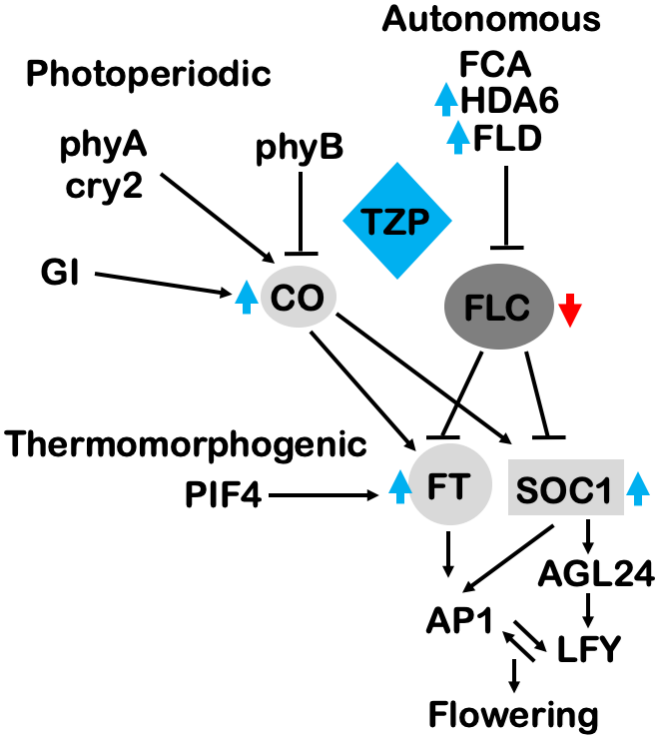
TANDEM ZINC‐FINGER/PLUS3 (TZP) is a positive regulator of flowering initiation by integrating light signaling and flowering pathways in Arabidopsis. TANDEM ZINC‐FINGER/PLUS3 induces the expression of key flowering regulators including *FT*, *CO* and *SOC1*. *FT* expression is induced via a direct association of TZP with the *FT* promoter (Kaiserli *et al*., 2015). Additionally, TZP indirectly contributes to the epigenetic silencing of the flowering repressor *FLC* by inducing the expression of the histone deacetylase *HDA6* and the histone demethylase *FLD* that in turn modulate the chromatin compaction of the *FLC* locus (Yu *et al*., 2011). AGL, AGAMOUS‐LIKE; AP1, APETALA 1; CO, CONSTANS; CRY, CRYPTOCHROME; FCA, FLOWERING TIME CONTROL A; FLC, FLOWERING LOCUS C; FLD, FLOWERING LOCUS D; FT, FLOWERING LOCUS T; GI, GIGANTEA; HDA6, HISTONE DEACETYLASE 6; LFY, LEAFY; PHY, PHYTOCHROME; PIF4, PHYTOCHROME INTERACTING FACTOR 4; SOC1, SUPPRESSOR OF OVEREXPRESSION OF CONSTANS 1; TZP, TANDEM ZINC‐FINGER/PLUS3. Red downward arrow defines downregulation; Blue upward arrow denotes upregulation; black line arrow indicates activation; black blunt‐ended arrows denote inhibition.

As previously mentioned, TZP was shown to downregulate the expression of *FLC* (Fig. 3c), but no TZP association was observed with the *FLC* locus based on ChIP‐seq analysis. However, our data showed that TZP binds to the promoters and induces the expression of the autonomous pathway components *FLD* and *HDA6* (Figs 4a,b,d,e, S5E) known to repress the expression of *FLC* through modulating its chromatin environment (Yu *et al*., 2011). Our study unveils that TZP indirectly influences the epigenetic status of *FLC* by increasing repressive histone marks on the *FLC* locus, while *tzp‐1* displays an increase in active histone marks including H3K9K14 acetylation and H3K4 methylation (Figs 5, 7).

The epigenetic regulation of *FLC* is well‐established (Whittaker & Dean, 2017), but our knowledge of how the chromatin‐remodeling complexes influencing *FLC* expression are regulated is very limited. We have uncovered that TZP plays a role in controlling flowering time by modulating the expression of key histone‐modifying enzymes and therefore regulating gene expression at the epigenetic level with respect to *FLC*. Whether TZP controls the epigenetic status of other flowering or growth‐promoting factors remains to be established.

Although TZP has been reported to act as a positive regulator of gene expression for targets related to growth (Perrella *et al*., 2018) and flowering (Kaiserli *et al*., 2015), the presence of a putative EAR motif known to recruit transcriptional corepressors in plants (Kagale & Rozwadowski, 2011) suggests an alternative function for TZP as a negative regulator of gene expression. Furthermore, TZP contains a PLUS3 domain with functions in cotranscriptional splicing, transcriptional elongation and chromatin remodeling in yeast and human proteins (de Jong *et al*., 2008; Wier *et al*., 2013; Cao *et al*., 2015). More specifically, the yeast PLUS3 domain containing protein Rtf1 is part of the Paf1 complex, which regulates histone ubiquitination, methylation and RNA 3′ end processing (de Jong *et al*., 2008; Wier *et al*., 2013; Cao *et al*., 2015). The Arabidopsis Paf1 complex induces *FLC* expression through H3 methylation (Yu & Michaels, 2010). Since TZP contains a PLUS3 domain and represses *FLC* through epigenetic regulation, it would be informative to assess whether TZP interacts with components of the Paf1 complex. Furthermore, TZP is known to interact with phyB (Kaiserli *et al*., 2015; Fang *et al*., 2022), and PHY is reported to facilitate the formation of a repressive chromatin loop by interacting with a component of the Polycomb Repressing complex2 (PRC2), VERNALIZATION‐INSENSITIVE 3‐LIKE1/VERNALIZATION 5 (VIL1/VRN5), leading to the repression of growth‐promoting genes including *ATHB2* (Kim *et al*., 2021). Since VIL1 is involved in controlling *FLC* repression and flowering, it would be interesting to investigate whether TZP operates in the same complex as phyB and VIL1 and whether they play a role in the vernalization pathway.

Overall, this study identifies a molecular mechanism in which TZP promotes flowering by indirectly controlling the silencing of the flowering repressor *FLC* that operates through the autonomous pathway (Fig. 7). In conclusion, these findings further unravel how light and temperature signals are perceived and integrated to obtain optimal plant growth and development and support the idea of TZP as a multifaceted transcriptional integrator of not only environmental but also endogenous stimuli.

## Competing interests

None declared.

## Author contributions

EK and GP directed the research and designed the experiments. GP and EV performed the experiments and analyzed the data. GH, AB and PH performed next‐generation sequencing and bioinformatics analysis. GP, GH, EV and EK wrote the manuscript. GP and EV contributed equally to this work.

## Disclaimer

The New Phytologist Foundation remains neutral with regard to jurisdictional claims in maps and in any institutional affiliations.

## Supporting information


**Fig. S1** Representative images of flowering assays shown in Fig. 1.
**Fig. S2** Plots visualizing differentially expressed genes of the RNA‐seq data shown in Fig. 2.
**Fig. S3** RNA‐seq data analysis on Col‐0 and OXTZP.
**Fig. S4** Gene ontology analysis of differentially regulated genes.
**Fig. S5** Identification of promoter motifs and between ChIP‐seq and RNA‐seq TANDEM ZINC‐FINGER/PLUS3 targets.
**Fig. S6** Control of the methylation and acetylation status of the *TEMPRANILLO 1* locus by TANDEM ZINC‐FINGER/PLUS3.
**Fig. S7** Flowering assays and gene expression analysis of genetic crosses between OXTZP and FLOWERING LOCUS T.
**Table S1**. List of primers used in this study.

**Table S2** Comparison between Col‐0 and *tzp‐1* RNA‐seq datasets at Zeitgeber 0.5 associated with Fig. 2.

**Table S3** Comparison between Col‐0 and *tzp‐1* RNA‐seq datasets at Zeitgeber 15 associated with Fig. 2.

**Table S4** Gene ontology analysis of the upregulated genes between Col‐0 and *tzp‐1* identified by RNA‐seq at Zeitgeber 0.5 associated with Fig. S4.

**Table S5** Gene ontology analysis of the downregulated genes between Col‐0 and *tzp‐1* identified by RNA‐seq at Zeitgeber 0.5 associated with Fig. S4.

**Table S6** Gene ontology analysis of the upregulated genes between Col‐0 and *tzp‐1* identified by RNA‐seq at Zeitgeber 15 associated with Fig. S4.

**Table S7** Gene ontology analysis of the downregulated genes between Col‐0 and *tzp‐1* identified by RNA‐seq at Zeitgeber 15 associated with Fig. S4.

**Table S8** Comparison between Col‐0 Zeitgeber 0.5 and 15 RNA‐seq datasets associated with Fig. S3.

**Table S9** Comparison between Col‐0 and OXTZP RNA‐seq datasets at Zeitgeber 0.5 associated with Fig. S3.

**Table S10** Comparison between Col‐0 and OXTZP RNA‐seq datasets at Zeitgeber 15 associated with Fig. S3.

**Table S11** Comparison between OXTZP and TANDEM ZINC‐FINGER/PLUS3 RNA‐seq datasets at Zeitgeber 0.5 associated with Fig. S3.

**Table S12** Comparison between OXTZP and TANDEM ZINC‐FINGER/PLUS3 RNA‐seq datasets at Zeitgeber 15 associated with Fig. S3.

**Table S13** Gene ontology analysis of the upregulated genes between Col‐0 and OXTZP identified by RNA‐seq at Zeitgeber 0.5 associated with Fig. S4.

**Table S14** Gene ontology analysis of the downregulated genes between Col‐0 and OXTZP identified by RNA‐seq at Zeitgeber 0.5 associated with Fig. S4.

**Table S15** Gene ontology analysis of the upregulated genes between Col‐0 and OXTZP identified by RNA‐seq at Zeitgeber 15 associated with Fig. S4.

**Table S16** Gene ontology analysis of the downregulated genes between Col‐0 and OXTZP identified by RNA‐seq at Zeitgeber 15 associated with Fig. S4.

**Table S17** TANDEM ZINC‐FINGER/PLUS3 targets identified by ChIP‐seq at Zeitgeber 0.5 and 15 associated with Figs 4, S5.

**Table S18** Overlap of TANDEM ZINC‐FINGER/PLUS3 targets identified by RNA‐seq and ChIP‐seq at Zeitgeber 0.5 and 15 associated with Fig. S5.
Please note: Wiley is not responsible for the content or functionality of any Supporting Information supplied by the authors. Any queries (other than missing material) should be directed to the *New Phytologist* Central Office.

## Data Availability

Accession numbers associated with this work are as follows: TZP (TANDEM ZINC‐FINGER/PLUS3) AT5G43650; FLC (FLOWERING LOCUS C) AT5G10140; HDA6 (HISTONE DEACETYLASE 6) AT5G63110; FLD (FLOWERING LOCUS D) AT3G10390. Transcriptome (RNA‐seq) and genome‐wide sequencing (ChIP‐seq) data and analysis have been submitted to the European Nucleotide Archive (https://www.ebi.ac.uk/ena/browser/view/PRJEB87658) as Project no. PRJEB87658.
