# Supplementary material for: TANDEM ZINC‐FINGER/PLUS3 integrates light signaling and flowering regulatory pathways at the chromatin level
Source: New Phytol. 2025 May 12;247(2):706–18. doi: 10.1111/nph.70213 (PMC12177312; doi:10.1111/nph.70213)
Supplement: Supplementary file 1 — Fig. S1 Representative images of flowering assays shown in Fig. 1. Fig. S2 Plots visualizing differentially expressed genes of the RNA‐seq data shown in Fig. 2. Fig. S3 RNA‐seq data analysis on Col‐0 and OXTZP. Fig. S4 Gene ontology analysis of differentially regulated genes. Fig. S5 Identification of promoter motifs and between ChIP‐seq and RNA‐seq TANDEM ZINC‐FINGER/PLUS3 targets. Fig. S6 Control of the methylation and acetylation status of the TEMPRANILLO 1 locus by TANDEM ZINC‐FINGER/PLUS3. Fig. S7 Flowering assays and gene expression analysis of genetic crosses between OXTZP and FLOWERING LOCUS T. Table S1. List of primers used in this study. [file NPH-247-706-s012.pdf]

New Phytologist Supporting Information

Article title: TANDEM ZINC-FINGER/PLUS3 integrates light signaling and flowering regulatory pathways at the chromatin level

Authors: Giorgio Perrella, Elisa Vellutini, Allan Beveridge, Graham Hamilton, Pawel Herzyk and Eirini Kaiserli

Article acceptance date: 21 April 2025

Fig. S1

B

A

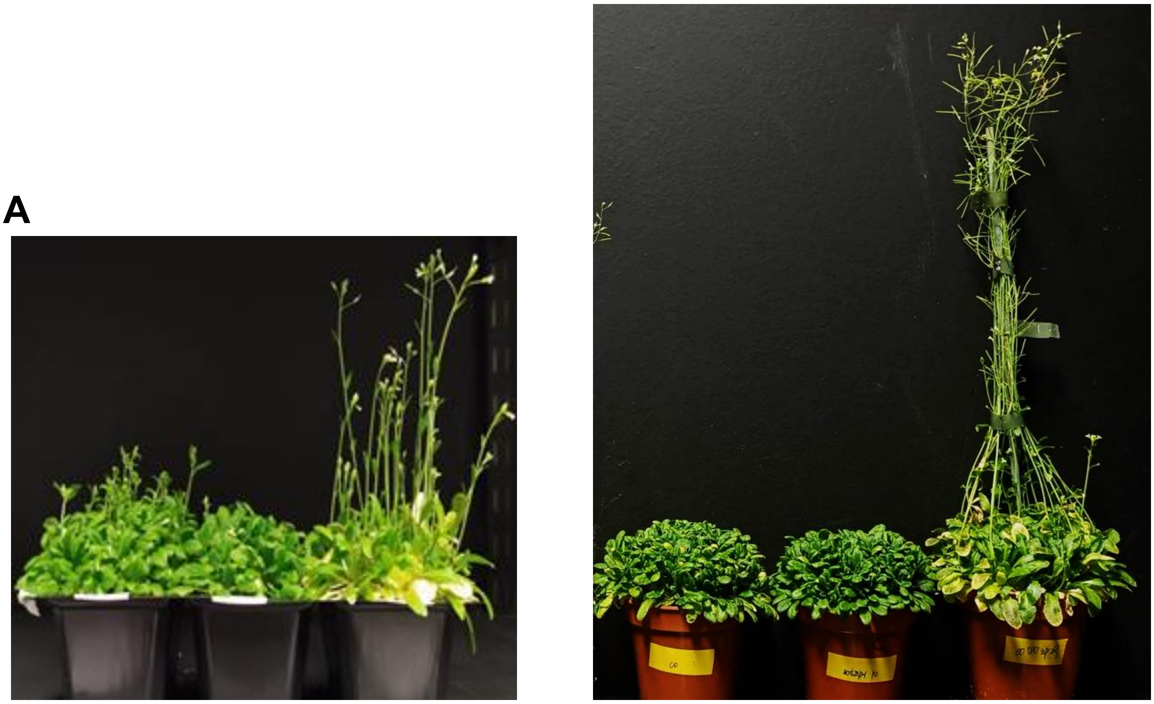

WT ***tzp-1*** **OXTZP**

co ***co/tzp-1*** ***co*/OXTZP**

Fig. S1 TZP promotes flowering initiation in a CONSTANS independent manner. Representative images of flowering time in (A) Col-0 (WT), *tzp-1,* OXTZP, and (B) *co*, *co/tzp-1* and *co*/OXTZP. OXTZP: Overexpression of TANDEM ZINC-FINGER/PLUS3; CO: CONSTANS.

Fig. S2

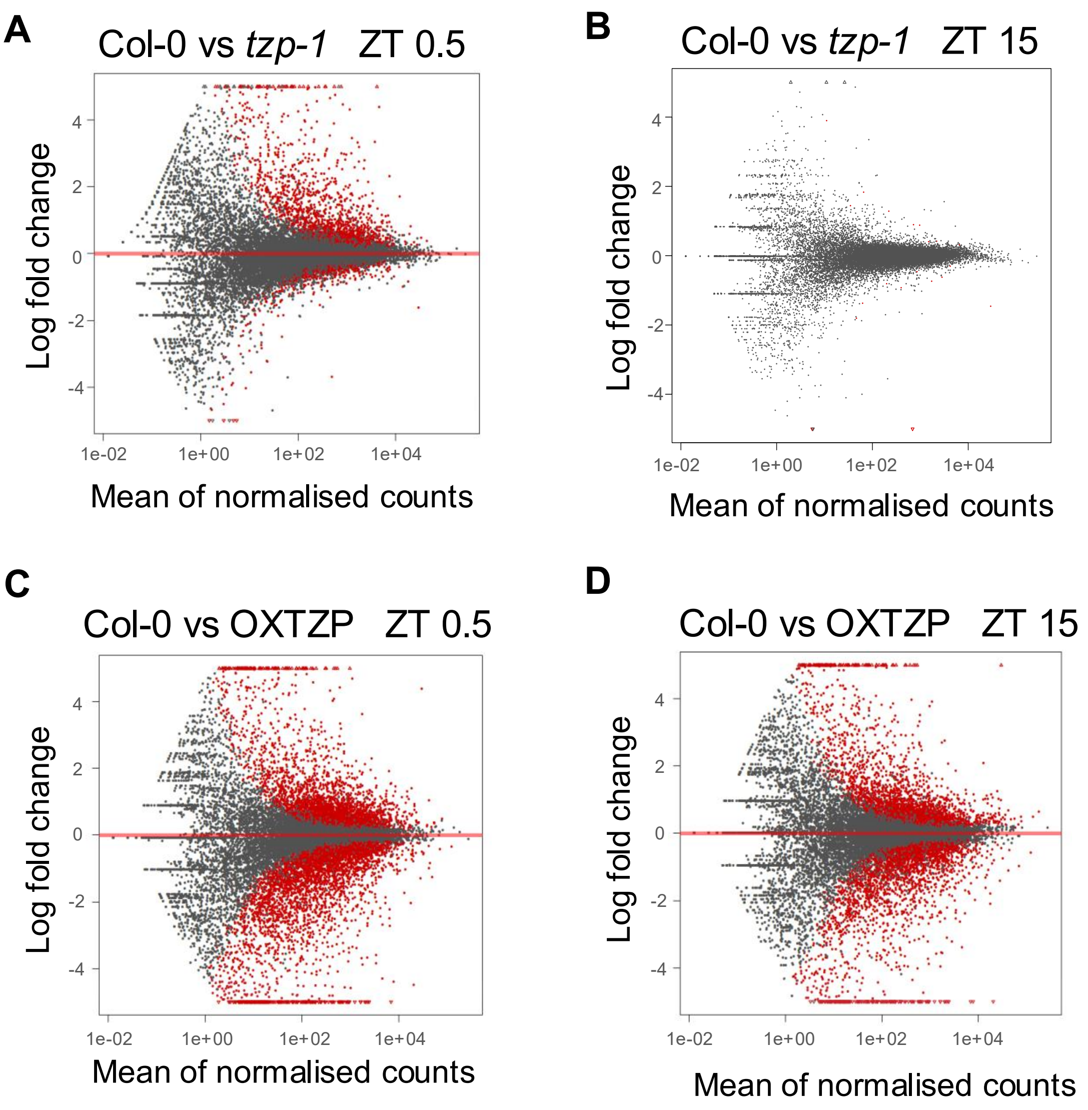

Fig. S2. (A-D) Plots visualizing differentially expressed genes where red dots indicate significant fold changes in gene expression (p-value <0.05) of Col-0 and *tzp-1* at ZT 0.5 (A) and ZT 15 (B) and Col-0 versus OXTZP at ZT 0.5 (C) and ZT 15 (D). OXTZP: Overexpression of TANDEM ZINC-FINGER/PLUS3; Log: logarithmic; ZT: Zeitgeber Time.

Fig. S3

A

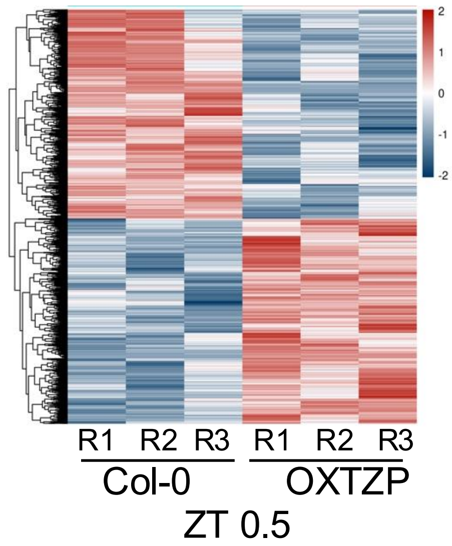

B

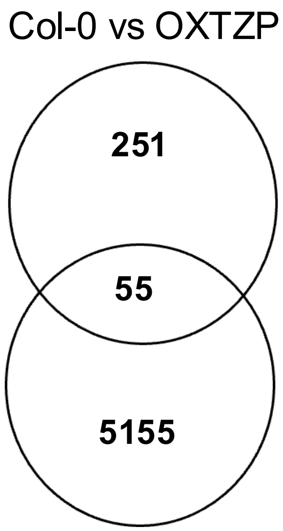

C

TCP18
COL9
CIB5
CDF4
CDF5
FD
FIO1
FT
FUL
GA1
GA20OX1
GA3OX1
JMJ30
MAF5
MYB30
OTS2
PRR3
PRR5
RVE2
AFR2
SPL3
SPL9
SUVR5
SUS4
SUF4
SOC1
ATX2
UGT87A2
FLX

GA3OX2
GLK1
HTA8
LNK1
PIF4
RUP2
RGL2
SMZ
SEF
SPL4
TEM1
VIM1
AGL15
AGL17
ATC
AHL22
STUBL4
CIB2
CLF
FKF1
BHLH122
FLC
FRI
GID1B
GA2
GASA5

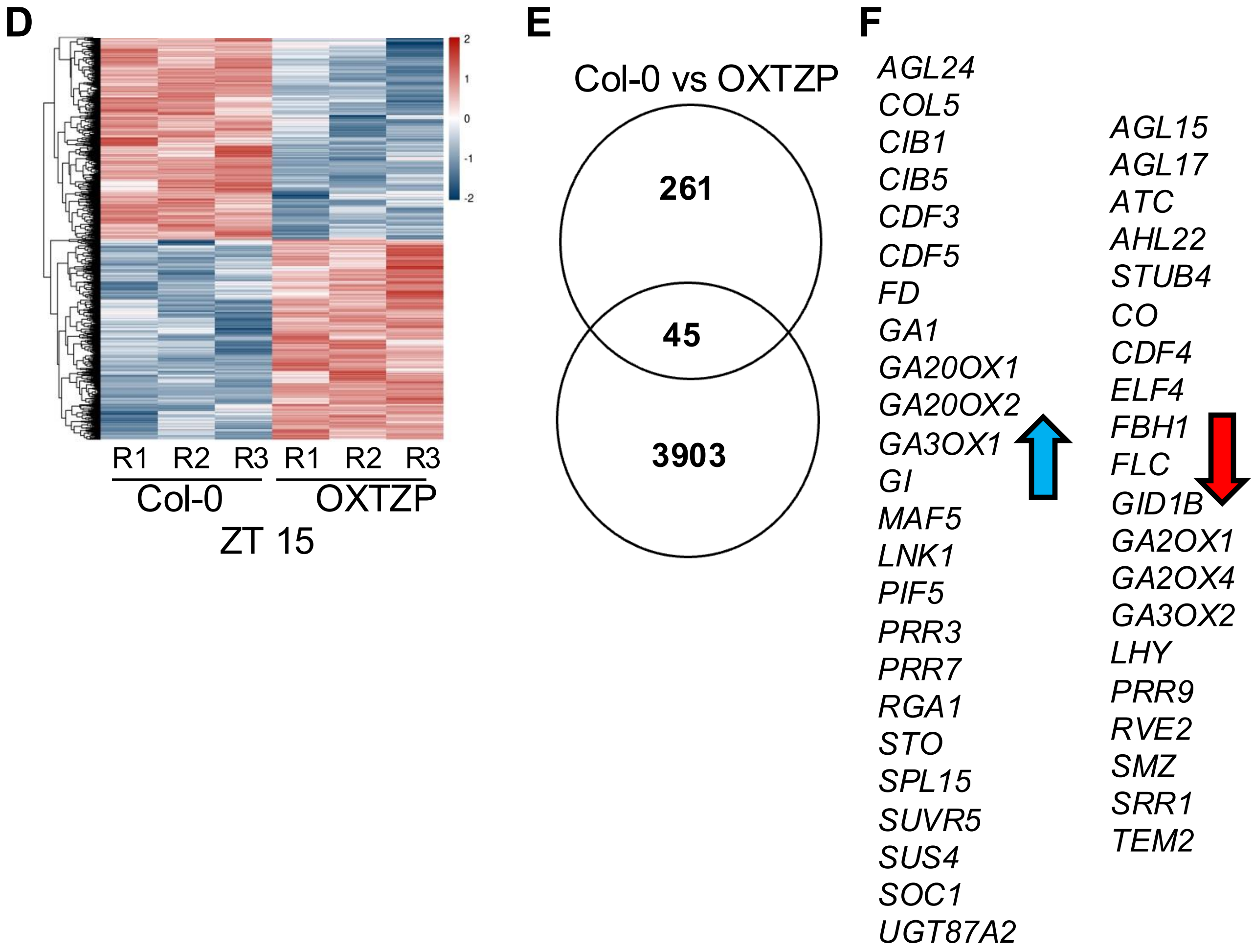

Fig. S3. **Hierarchical clustering of differentially expressed genes between the indicated genotypes**. Heatmaps depict the expression profile of differentially regulated genes between Col-0 and OXTZP at ZT 0.5 (A) and ZT 15 (D) (p-value <0.05; the colour shades depict the Z-score expression values, with blue as lower expression and red as higher; R1,2,3 depict the results from three biological replicates). Differentially expressed genes identified when comparing Col-0 and OXTZP at ZT 0.5 (B-C) and ZT 15 (E-F) were intersected with the list of flowering genes from the FLOR-ID database (Bouche et al., 2016). (B, E) Venn diagrams showing the overlap between differentially expressed genes between Col-0 and OXTZP (lower circle) with FLOR-ID (upper circle) at ZT 0.5 (B) and ZT 15 (D). (C, F) List of differentially regulated flowering genes from FLOR-ID database between Col-0 and OXTZP at ZT 0.5 (C) and ZT 15 (F). The blue up arrow indicates upregulated genes and the red down arrow indicates downregulated genes. Interpolation and Venn diagram were created using the online tool Venny 2.1 (bioinfogp.cnb.csic.es/tools/venny/). OXTZP: Overexpression of TANDEM ZINC-FINGER/PLUS3; ZT: Zeitgeber Time.

Fig. S4

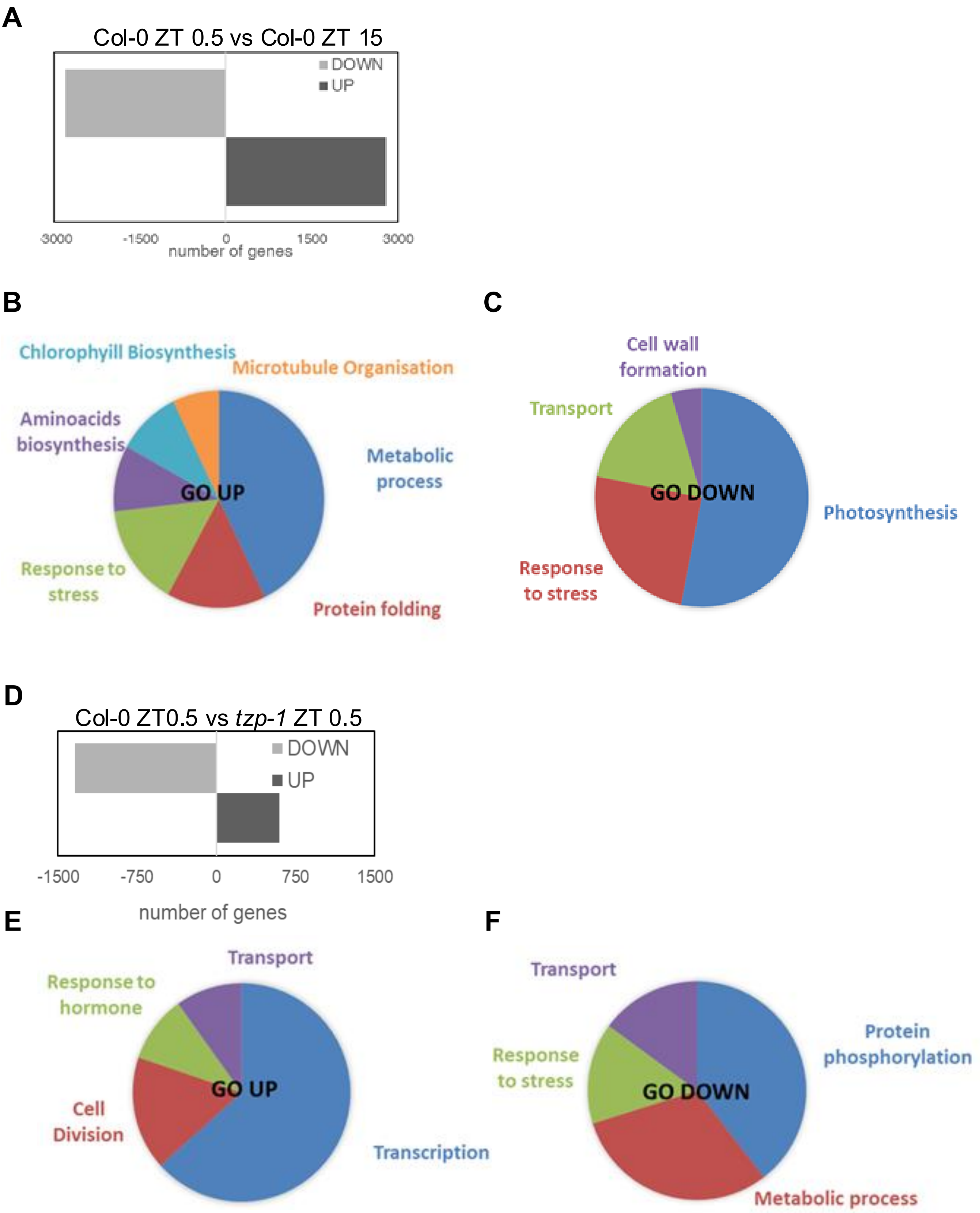
A
Col-0 ZT 0.5 vs Col-0 ZT 15
DOWN
UP
3000
-1500
0
1500
3000
number of genes
B
Chlorophyill Biosynthesis
Microtubule Organisation
Aminoacids biosynthesis
Metabolic process
GO UP
Response to stress
Protein folding
C
Cell wall formation
Transport
GO DOWN
Photosynthesis
Response to stress
D
Col-0 ZT0.5 vs tzp-1 ZT 0.5
DOWN
UP
-1500
-750
0
750
1500
number of genes
E
Transport
Response to hormone
GO UP
Cell Division
Transcription
F
Transport
Protein phosphorylation
Response to stress
GO DOWN
Metabolic process

G

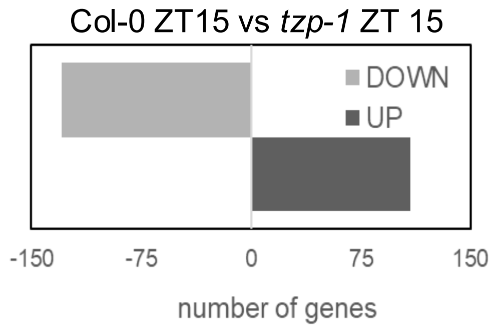
Col-0 ZT15 vs *tzp-1* ZT 15
DOWN
UP
-150
-75
0
75
150
number of genes

H

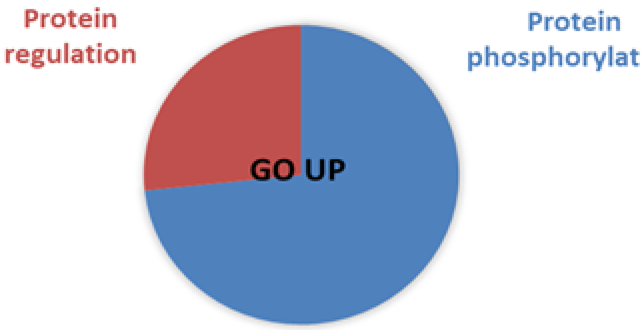
Protein regulation
Protein phosphorylat
GO UP

I

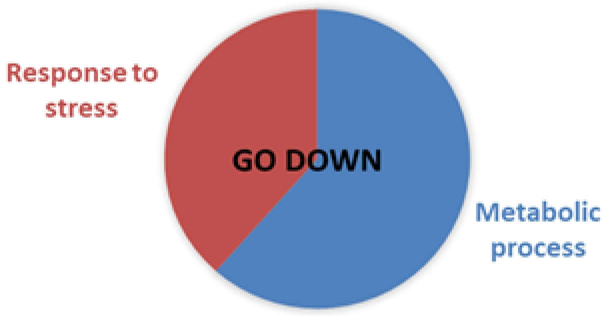
Response to stress
GO DOWN
Metabolic process

J

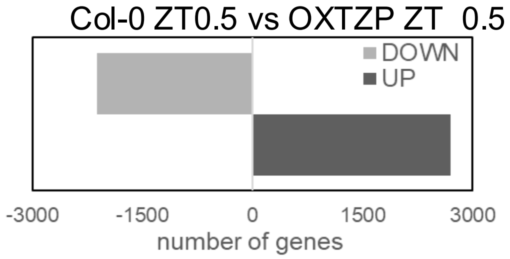
Col-0 ZT0.5 vs OXTZP ZT 0.5
DOWN
UP
-3000
-1500
0
1500
3000
number of genes

K

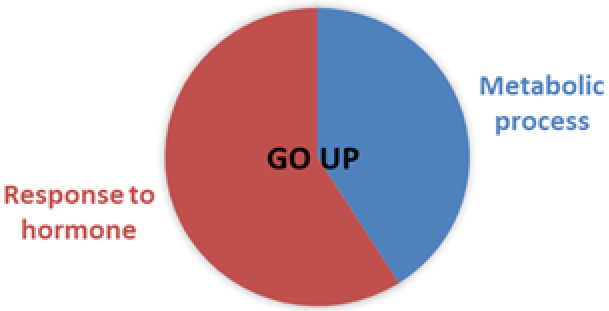
Metabolic process
GO UP
Response to hormone

L

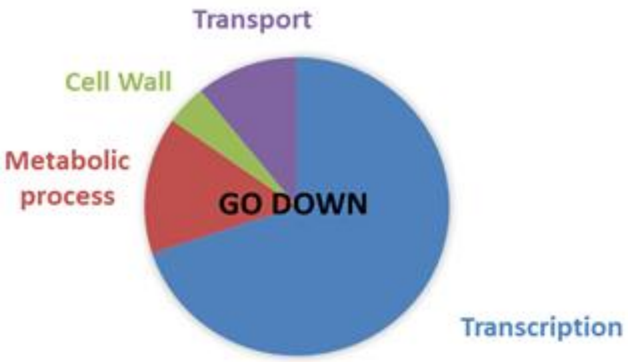
Transport
Cell Wall
Metabolic process
GO DOWN
Transcription

M

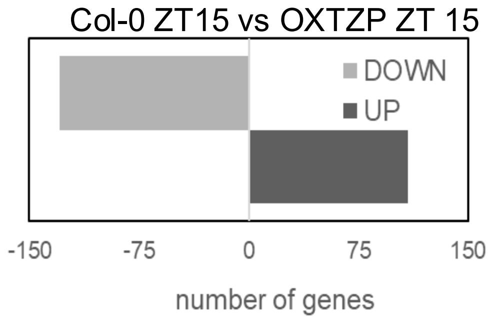

N

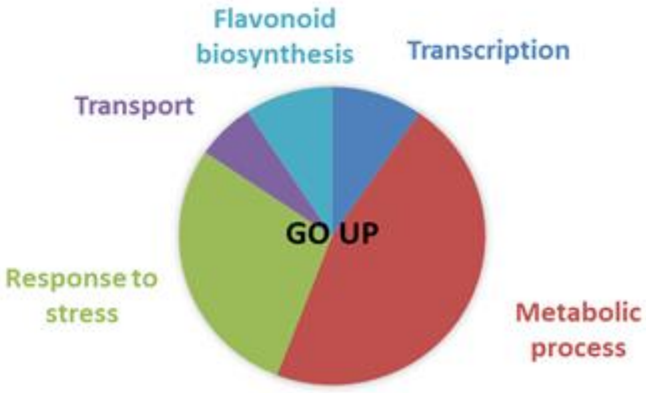

O

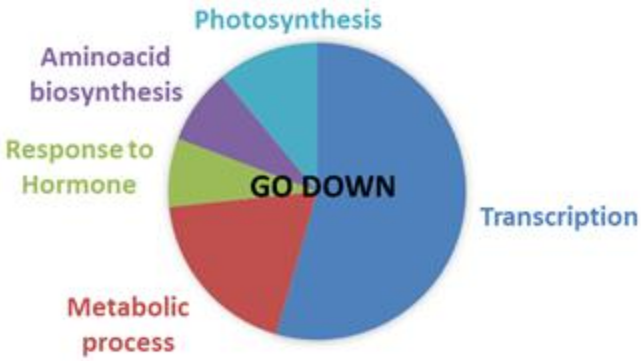

Fig. S4. TZP regulates gene expression in a time of the day independent manner. (A, D, G, M) Bar graphs depicting differentially down and up-regulated genes for the corresponding genotypes and conditions (1.5 fold change and p value of 0.05). (B-C, E-F, H-I, N-O) Functional characterization of gene loci up and downregulated, respectively. Enrichment analysis based on Biological Process was performed using the gene ontology (GO) TERM DIRECT in DAVID functional annotation chart. OXTZP: Overexpression of TANDEM ZINC-FINGER/PLUS3; ZT: Zeitgeber Time.

Fig. S5

A

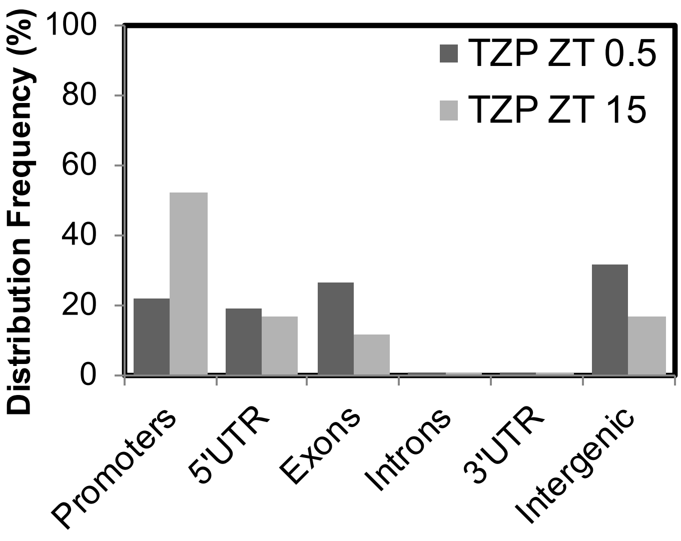

B

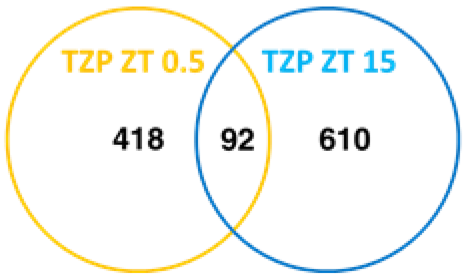

C

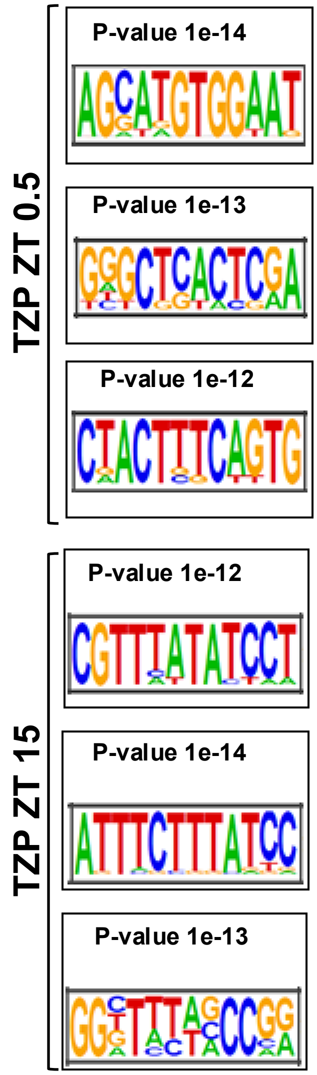

D

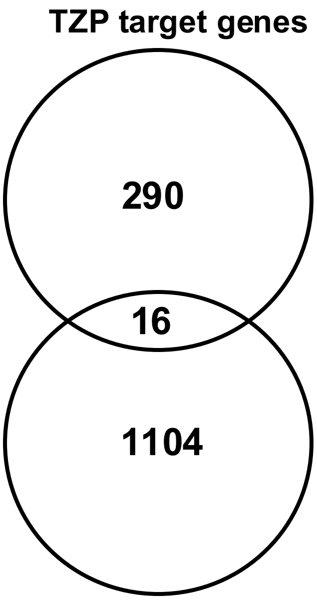

E

Flowering Genes

AP1
AP2
CCA1
FBH4
FLD
GAI
GA2OX7
HDA6
LHY
MYB30
PIF5
RCD1
RVE2
SDG26
SOC1
TEM1

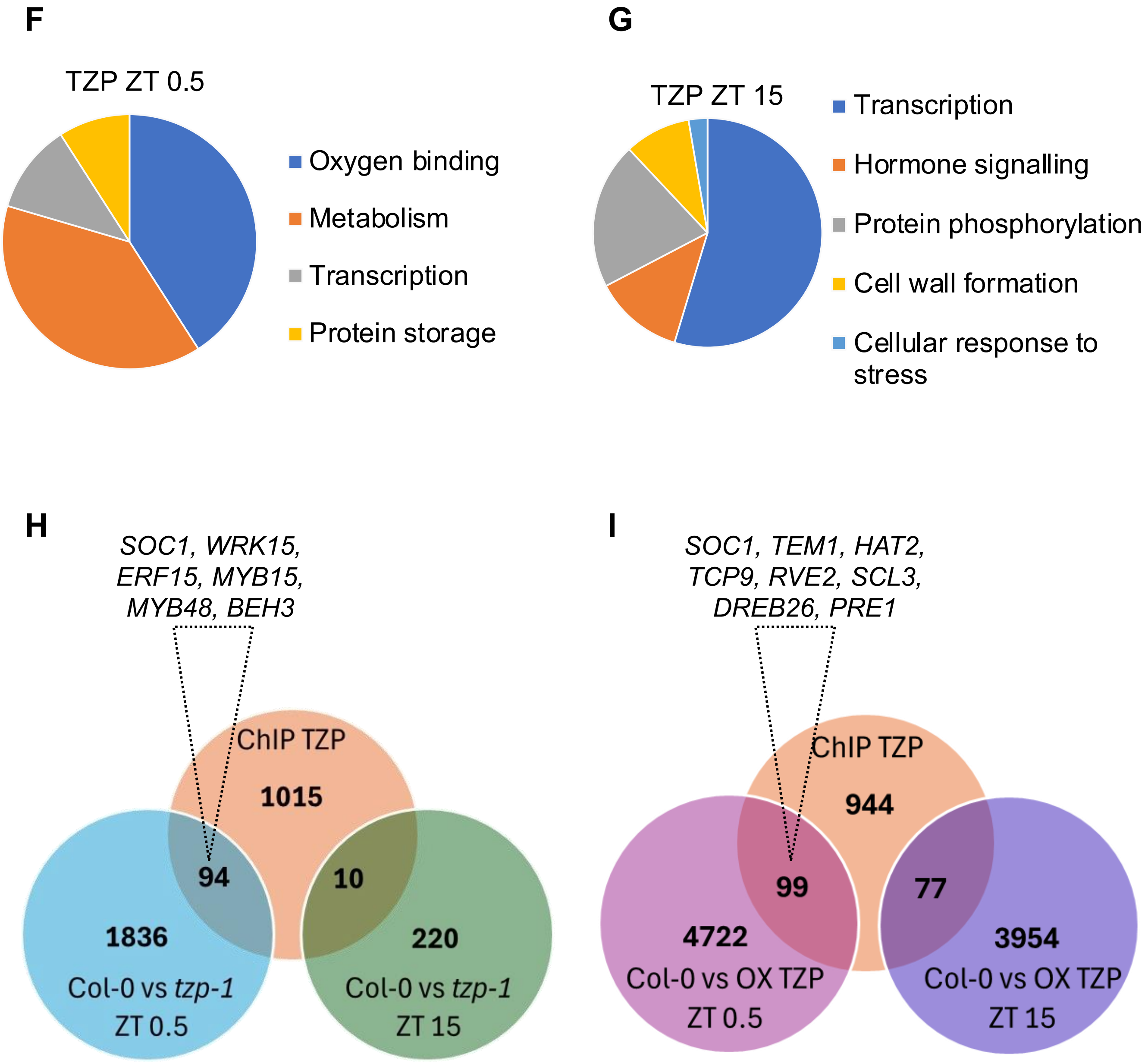
F
TZP ZT 0.5
Oxygen binding
Metabolism
Transcription
Protein storage
G
TZP ZT 15
Transcription
Hormone signalling
Protein phosphorylation
Cell wall formation
Cellular response to stress
H
SOC1, WRK15, ERF15, MYB15, MYB48, BEH3
ChIP TZP
1015
94
10
1836
220
Col-0 vs tzp-1 ZT 0.5
Col-0 vs tzp-1 ZT 15
I
SOC1, TEM1, HAT2, TCP9, RVE2, SCL3, DREB26, PRE1
ChIP TZP
944
99
77
4722
3954
Col-0 vs OX TZP ZT 0.5
Col-0 vs OX TZP ZT 15

Fig. S5. ChIP-seq reveals TZP binding elements during the day. (A) Relative binding-peak distribution of TZP at ZT 0.5 and ZT 15 across genomic regions. Values are normalised to those of Col-0. (B) Venn diagram depicting the overlap of TZP binding at ZT 0.5 and ZT 15 (promoter and TSS). (C) Enrichment for motifs identified within 1Kb regions for TZP at ZT 0.5 and ZT 15. (D) Overlap between TZP target genes and flowering genes from FLOR-ID database. Venn diagram of TZP target genes identified with ChIP-seq at ZT 0.5 and ZT 15 and intersected with the pool of flowering genes from the FLOR-ID database. (E) The genes listed are both targets of TZP and flowering-related genes. Functional characterization of gene loci associated by TZP at ZT 0.5 (F) and ZT 15 (G). Enrichment analysis based on Biological Process was performed using the gene ontology (GO) TERM DIRECT in DAVID functional annotation chart. (H-I) Venn Diagrams depicting the overlap between TZP ChIP-seq targets and differentially expressed genes (1.5 fold change and p-value of 0.05) between Col-0 and *tzp-1* at ZT 0.5 and ZT 15 (H) and Col-0 and OXTZP at ZT 0.5 and ZT 15 (I). Close ups highlight the shared targets involved in flowering, circadian clock and response to hormones. . OXTZP: Overexpression of TANDEM ZINC-FINGER/PLUS3; ZT: Zeitgeber Time; TSS: Transcriptional Start Site; UTR: Untranslated Region.

Fig. S6

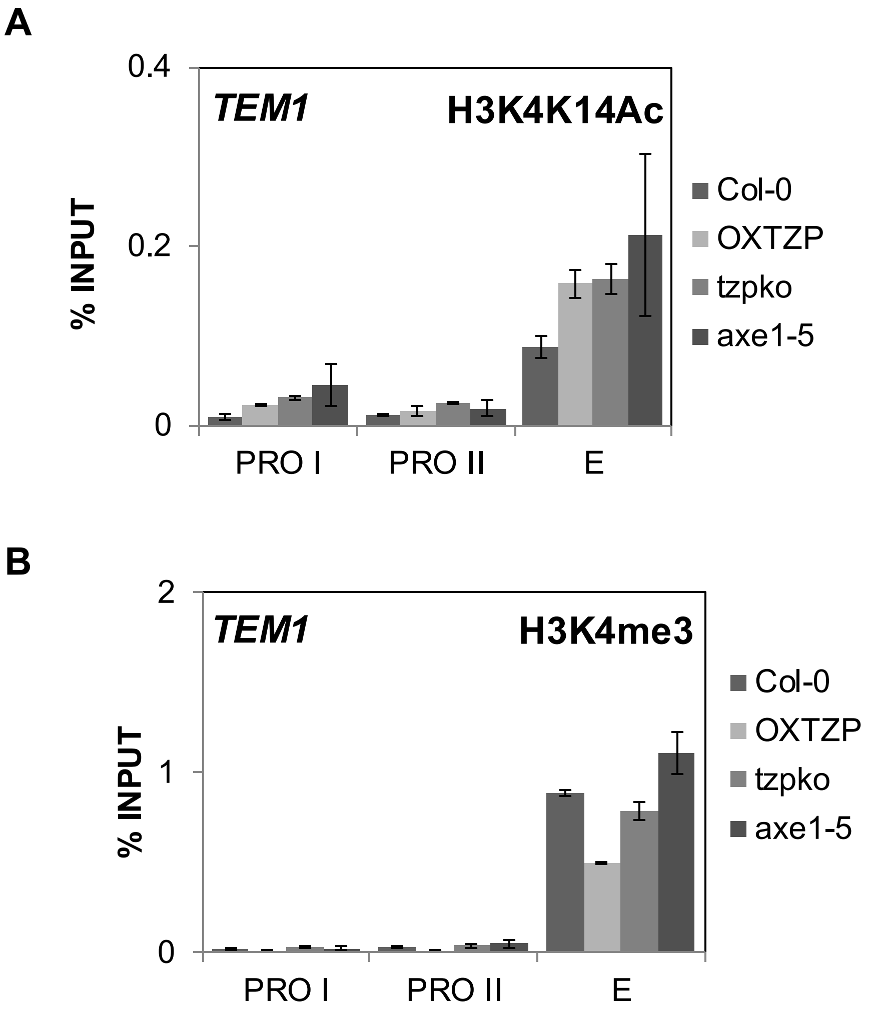

Fig. S6. TZP control of *TEM1* acetylation and methylation status. Relative H3K9K14Ac (A) and H3K4me3 (B) enrichment in Col-0, OX TZP, *tzp-1* and *axe1-5*, at ZT 0.5. Seedlings were grown in white light (75 mmol m^{-2} s^{-1}) for twelve days and samples were harvested at ZT 0.5 of day 12. PRO indicates promoter regions. . OXTZP: Overexpression of TANDEM ZINC-FINGER/PLUS3; ZT: Zeitgeber Time; KO: knockout; TEM1: TEMPRANILLO 1; axe1-5: *flowering locus d* mutant; PRO: promoter region.

Fig. S7

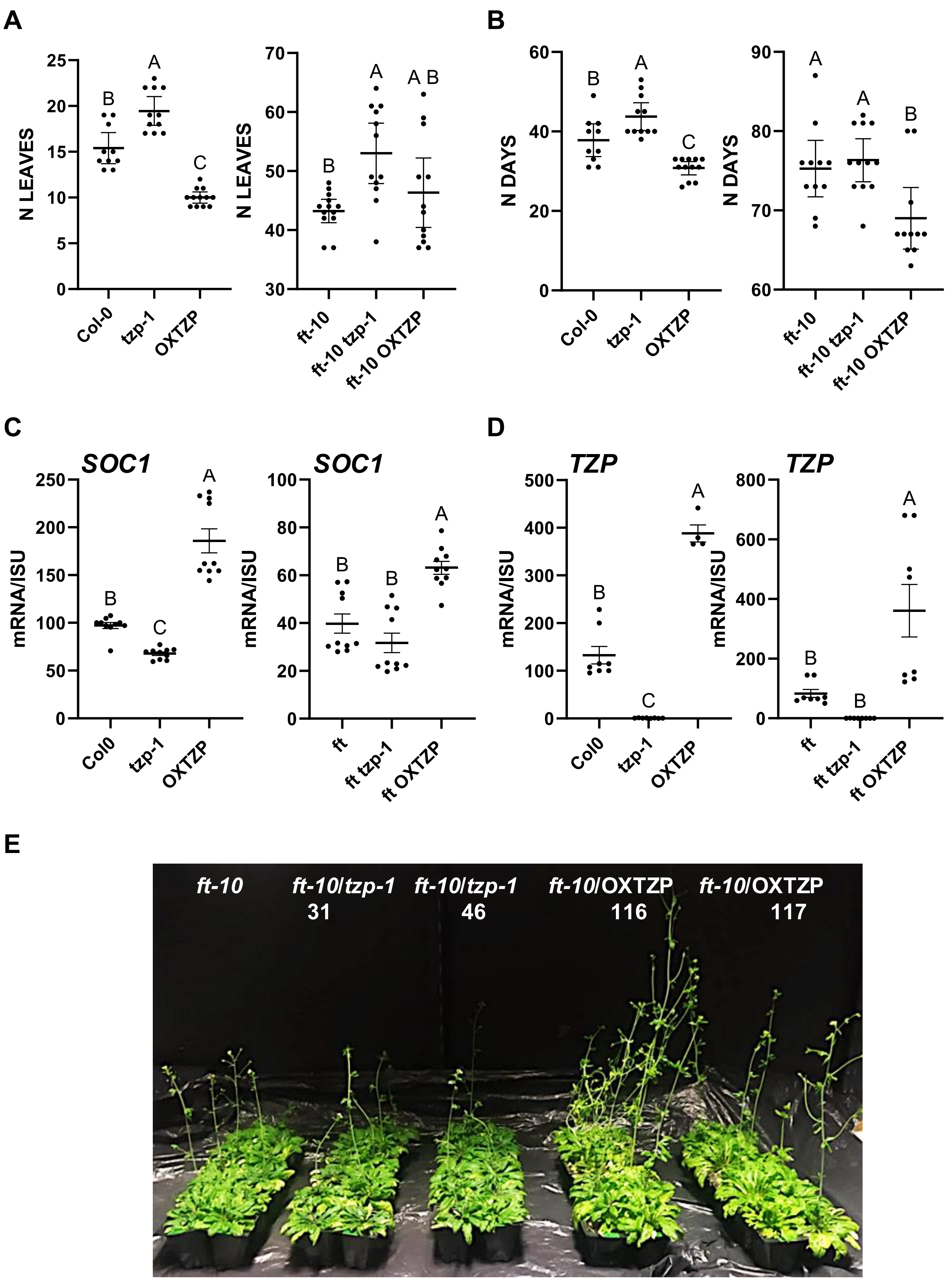
A
N LEAVES
Col-0
tzp-1
OXTZP
ft-10
ft-10 tzp-1
ft-10 OXTZP
B
N DAYS
C
SOC1
mRNA/ISU
Col0
ft
ft tzp-1
ft OXTZP
D
TZP
E
ft-10
ft-10/tzp-1
31
46
ft-10/OXTZP
116
117

Fig. S7. TZP overexpression partially rescues the late flowering phenotype of *ft-10*. (A-B) Phenotypic characterisation of flowering time in genetic crosses between *ft-10* and *tzp-1* or OXTZP. Plants were grown under long day LD 16h light/8h dark photoperiodic conditions. Data are represented as mean ± 95% confidence interval (n = 12 plants). Data are representative of 3 biological replicates. One-way ANOVA with Tukey's multiple comparison post hoc test was performed amongst the genotypes indicated per graph. Quantitively RT-PCR analysis of *SOC1* (C) and *TZP* (D) transcript levels normalised with housekeeping gene *ISU1*. Tissue was isolated at ZT 15 on day 12 under LD white light (50 µmol m^{-2} s^{-1}). The wild type (Col-0) was used as a reference. Data are represented as mean ± SEM. Data are representative of 4 technical repeats and two biological replicates. One-way ANOVA with Tukey's multiple comparison post hoc test was performed amongst the genotypes indicated per graph. (E) Representative image of the flowering phenotypes shown in (A-B). . OXTZP: Overexpression of TANDEM ZINC-FINGER/PLUS3; SOC1: SUPPRESSOR OF OVEREXPRESSION OF CONSTANS 1; FT: FLOWERING LOCUS T; ISU1: IRON-SULFUR CLUSTER ASSEMBLY PROTEIN 1.

Table S1 List of primers used in this study.

Primers used for genotyping.

Mutation genotyped	Primers for WT detection	Primers for Mutant detection
tzp-1	TZP_LP + TZP_RP	LBb1.3 + TZP_RP
ft-10	FT_LP + FT_RP	FT_LP + PAC161
co-10	CO_LP + CO_RP	LB3 + CO_RP
tzp-1 LP	ACGCTTCTTCTCTTCCATTCC	
tzp-1 RP	TGTTTGGGGTCAACTTCAAAG	
LBb1.3	ATTTTGCCGATTTCGGAAC	
LB3	TAGCATCTGAATTTCATAACCAATCTCGATACAC	
ft-10 LP	GGTGGAGAAGACCTCAGGAAC	
ft-10 RP	TTTTGGGAGACAAATTGATGC	
co-10 LP	AAGCTGTTGTGACACATGCTG	
co-10 RP	CCCCTTCTTTCAGATACCAGC	
flc-3-F	TATCGCCGGAGGAGAAGC	
flc-3-R	TAGAAAGAAATAAAGCGAGAAAAGGA	

Primers used for RT-qPCR.

TZP for	CCAAGACTTTCTTGAGGAGGAG
TZP rev	GCCGCTTGTTCTGGCACTT
ISU for	GCCATCGCTTCTTCATCTGTTGC
ISU rev	TGGGAGAGAAAGATGCTTTG CG
FT for	CTAGCAACCCTCACCTCCGAGAATA
FT rev	CTGCCAAGCTGTCGAAACAATATAA
FLC for	CCCGGGGTACAATCATCTCC
FLC rev	AGCTTCAACATGAGTTCGGTCTTC
HDA6 for	ACCTAGCGTCCAGTTTCAGC
HDA6 rev	AAGTCGCAGTTCCACTCCAG
FLD for	GAGACAATGCCACCCACTGA
FLD rev	TGAAGCTCCCACTGCAACAT

FT for	CTAGCAACCCTCACCTCCGAGAATA
FT rev	CTGCCAAGCTGTCGAAACAATATAA
FLC for	CCCGGGGTACAATCATCTCC
FLC rev	AGCTTCAACATGAGTTCGGTCTTC
SOC1 for	TCAATCGAGGAGCTGCAACA
SOC1 rev	CGCTTTCATGAGATCCCCACT
GI for	TTTCTCCGATATTCGTCGATCTC
GI rev	CTGAATCAAACAGCTAAACCC
FD for	CATCAACCTTGCTTCCATCC
FD rev	GGTTTTGGTTGTGGTGGTTT

Primers used for ChIP-qPCR

MAF4-P-F	GGTCGGTTTAGAGTCCAATC
MAF4-P-R	TGGTGTAAGATAGTTCCACG
MAF4-E-F	CGCACCGTTTAGACTCTTTG
MAF4-E-R	GTTGACGAGCTTTCTCCATG
MAF4-I-F	GTCTTGGGATTTCAAGCCATC
MAF4-I-R	TCTCAACAAATCTACCGGGG
MAF4-U-F	CATCACCAACGACTGATTCG
MAF4-U-R	TAGGGTTCTTCACGCTGAAG
MAF5-P-F	TTACATGGCTCAGCCCAATG
MAF5-P-R	AAAGGATTAGCCGAAGTGGG
MAF5-E-F	CTCAGGGAATTCAGCATGCG
MAF5-E-R	GGAGGATCCACAGAGAATTG
MAF5-I-F	CCTTGCATGCACTTCAAGAG
MAF5-I-R	GCACTCGTTTCCACTAGATTC
MAF5-U-F	AAAATAGCTCTGGCCACAGC
MAF5-U-R	TCTGATCCTGTCTTCCAAGG
SOC1-PRO-F	GCAAAAGAAGTAGCTTTCCTCG
SOC1-PRO-R	AGCAGAGAGAGAAGAGACGAGTG
SOC1-TSS-F	GGTAGATCAATGGTGCAAC
SOC1-TSS-R	CTTCATCAGTCTTCTCCCAC
SOC1-I-F	CCTGATTTTGACATAGAAACTC
SOC1-I-R	TCAAAAGTAAGTTTGGTTTCC

SOC1-UTR-F	GGTTTAGCAGTACTGAGAGTGTATAAGG
SOC1-UTR-R	GTTCAGAATCATTAAAGAAGTGACTGAG
TEM1-PRO-F	TTCTACGTACAAAGAAAGTGCTTAGGG
TEM1-PRO-R	TCCATATTTGGAACATGACGTG
TEM1-TSS-F	ATGAAGGGACTAATTATGGCAACA
TEM1-TSS-R	AAGTTTCGTGGGAAGAGTCCA
TEM1-EX-F	CGGTTCAGACTGTGGTTAGGC
TEM1-EX-R	AATCGCCTGCTTCTTGGAAC
FLC-P-F	TGTAGGCACGACTTTGGTAACACC
FLC-P-R	GCAGAAAGAACCTCCACTCTACATC
FLC-E-F	CGACTTGAACCCAAACCTGAGGATCAAAT
FLC-E-R	AGAAGATAAAAGGGGGAACAAATGAAAAC
FLC-I-F	CTGCGACCATGATAGATACATGAGA
FLC-I-R	TTCACTCAACAACATCGAGCACG
